# A novel approach to exploring the dark genome and its application to mapping of the vertebrate virus fossil record

**DOI:** 10.1186/s13059-024-03258-y

**Published:** 2024-05-13

**Authors:** Daniel Blanco-Melo, Matthew A. Campbell, Henan Zhu, Tristan P. W. Dennis, Sejal Modha, Spyros Lytras, Joseph Hughes, Anna Gatseva, Robert J. Gifford

**Affiliations:** 1 Vaccine and Infectious Disease Division, Fred Hutchinson Cancer Center, 1100 Fairview Ave N, Seattle, WA 98109 USA; 2 Herbold Computational Biology Program, Public Health Sciences Division, Fred Hutchinson Cancer Center, 1100 Fairview Ave N, Seattle, WA 98109 USA; 3 University of California, Davis, 1 Shields Ave, Davis, CA 95616 USA; 4 MRC-University of Glasgow Centre for Virus Research, 464 Bearsden Rd, Bearsden, Glasgow, G61 1QH UK; 5 Centre for Epidemic Response and Innovation (CERI), School of Data Science and Computational Thinking, Stellenbosch University, Stellenbosch, South Africa

## Abstract

**Background:**

Genomic regions that remain poorly understood, often referred to as the dark genome, contain a variety of functionally relevant and biologically informative features. These include endogenous viral elements (EVEs)—virus-derived sequences that can dramatically impact host biology and serve as a virus fossil record. In this study, we introduce a database-integrated genome screening (DIGS) approach to investigate the dark genome in silico, focusing on EVEs found within vertebrate genomes.

**Results:**

Using DIGS on 874 vertebrate genomes, we uncover approximately 1.1 million EVE sequences, with over 99% originating from endogenous retroviruses or transposable elements that contain EVE DNA. We show that the remaining 6038 sequences represent over a thousand distinct horizontal gene transfer events across 10 virus families, including some that have not previously been reported as EVEs. We explore the genomic and phylogenetic characteristics of non-retroviral EVEs and determine their rates of acquisition during vertebrate evolution. Our study uncovers novel virus diversity, broadens knowledge of virus distribution among vertebrate hosts, and provides new insights into the ecology and evolution of vertebrate viruses.

**Conclusions:**

We comprehensively catalog and analyze EVEs within 874 vertebrate genomes, shedding light on the distribution, diversity, and long-term evolution of viruses and reveal their extensive impact on vertebrate genome evolution. Our results demonstrate the power of linking a relational database management system to a similarity search-based screening pipeline for in silico exploration of the dark genome.

**Supplementary Information:**

The online version contains supplementary material available at 10.1186/s13059-024-03258-y.

## Introduction

The availability of whole genome sequence (WGS) data from a broad range of species provides unprecedented scope for comparative genomic investigations [1–3]. However, these investigations rely to a large extent on *annotation*—the process of identifying and labeling genome features—which usually lags far behind the generation of sequence data. Consequently, most whole genome sequences are comprised of DNA that is incompletely understood in terms of its evolutionary origins and functional significance. The portion of sequenced genome space that lacks annotations is sometimes referred to as the “dark genome” [4] and contains a wide variety of yet-to-be-characterized genome features. Some of these may have functional roles, such as encoding proteins [5] or regulating gene expression [6]. Others, such as non-expressed pseudogenes, may not but can nonetheless provide valuable insights into genome biology and evolution.

Within the dark genome, endogenous viral elements (EVEs) constitute a particularly intriguing group of genome features. EVEs are virus-derived DNA sequences that become integrated into the germline genome of host species and are stably inherited as host alleles—a form of horizontal gene transfer [7–14]. While once considered genetic “junk”, it has become evident over recent years that EVEs can profoundly impact host biology and genome evolution, with many now known to have physiologically relevant roles [15–19]. In addition, EVE sequences (whether functional or not) provide a rare source of retrospective information about ancient viruses, akin to a viral “fossil record” [7, 20–22].

**Fig. 1:**
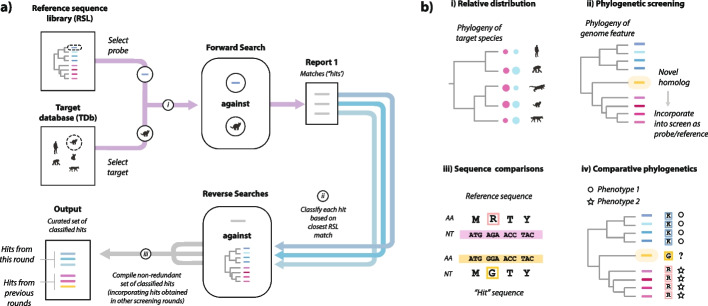
Exploring the dark genome using in silico screening. **a** Overview of sequence similarity search-based screening. Screening aims to identify and classify sequences similar to a set of query sequences within a target database (TDb) comprising whole genome sequence assemblies of multiple species. The schematic shows the steps that comprise a single round of screening, as follows: (i) a BLAST search is performed using a probe sequence selected from a curated “reference sequence library” (RSL) and a “target” file is selected from the TDb; (ii) matching sequences (referred to as “hits”) identified in this screen are classified via similarity search-based comparison to the RSL; and (iii) a non-redundant set of classified hits is compiled, incorporating hits from previous rounds of screening. **b** Comparative analysis of screen output. Sequences recovered via screening can be investigated using a wide range of comparative approaches, as follows: (i) analysis of feature distribution—e.g., annotating host phylogeny to show frequency of occurrence (colored circles); (ii) phylogenetic screening, in which sequences obtained via similarity search-based screening are investigated in phylogenetic reconstructions (e.g., to identify novel lineages not present in the RSL, as shown here); (iii) pairwise sequence comparisons—these can be used to identify differences in sequences obtained via screening, relative to reference sequences; and (iv) comparative phylogenetic analysis—the genetic properties of novel homologs can be inferred via comparative analysis (e.g., pairwise comparisons), while their phenotypic properties can potentially be investigated experimentally (e.g., via transcriptome sequencing)

Identifying genome features contained within the dark genome, such as EVEs, often relies on the use of sequence similarity searches, such as those implemented in the Basic Local Alignment Search Tool (BLAST) [23, 24], to search WGS databases. Because sequence similarity reflects homology (evolutionary relatedness), novel genome features can often be identified based on their resemblance to ones that have been described previously. One example of this approach is implemented in the PSI-BLAST [5] and HMMR [8] programs, in which iterated search strategies are used to progressively increase sensitivity so that novel homologs of previously characterized genes may be detected. A related approach is “systematic in silico genome screening” which extends the basic concept of a similarity search in two ways: (i) inclusion of multiple query sequences and/or target databases and (ii) similarity-based classification of matching sequences (“hits”) via comparison to a reference sequence library (Fig. 1a). Hits may also be further investigated using additional comparative or experimental approaches (Fig. 1b, Table 1). Thus, screening can provide one component of a broader analytical pipeline.

**Table 1:** Examples of published studies utilizing database-integrated screening

**Genome feature**	**Target database**	**Reference sequence library and probes**^**c**^	**Reference**	**Year**
**Non-coding DNA**				
ZP3AR (and SFP819)	Rodents	ZP3AR^c^, ZFP819^c^, and related genes	[25]^b^	2022
SHIN (and IAP elements)	Rodents	SHIN^c^, IAP subgroups^c^, Retroviridae	[26]^b^	2023
**Genes**				
OAS1 gene	Mammals	OAS1^c^ and related genes	[27]^b^	2021
APOBEC3 (A3) genes	Mammals	APOBEC3^c^ and related genes	[28]^a^	2020
Interferon stimulated genes (ISGs)	Vertebrates	ISGs^c^ and related genes	[29]	2017
Interferon lambda (IFNL) genes	Vertebrates	IFNLs^c^ and locus marker genes^c^	[30]^a,b^	2023
**Endogenous viral elements**				
Family Flaviviridae	Metazoa	AVP, Flaviviridae^c^, and EFVs	[31]^a^	2022
Family Parvoviridae	Vertebrates	AVP, Parvoviridae^c^, and EPVs	[32]^a^	2022
Family Parvoviridae	Vertebrates	AVP, Parvoviridae^c^, and EPVs	[33]^b^	2023
Genus Protoparvovirus	Mammals	AVP, protoparvoviruses^c^, and EPVs	[34]^a,b^	2019
Family Hepadnaviridae	Metazoa	AVP, Hepadnaviridae^c^, and eHBVs	[35]^a^	2021
Family Circoviridae	Metazoa	AVP, Circoviridae^c^, and ECVs	[36]^a^	2019
**Endogenous retroviruses**				
Genus *Lentivirus*	Rodents	Lentiviruses^c^, other XRVs, & ERVs	[37]^a^	2022
Family Retroviridae	Perissodactyls	Retroviridae^c^, Retroelements	[38]^a^	2018
HERV-T	Hominids	Class I HERVs^c^, Retroviridae	[39]^a,b^	2017
MuERV-L	Mice	Class III ERVs^c^, Retroviridae	[40]^b^	2018

*ZFP* Zinc finger protein, *OAS1* 2’-5’-oligoadenylate synthetase 1, *IAP* Intracisternal A-type particle, *EFV* Endogenous flaviviral element, *EPV* Endogenous parvoviral element, *eHBV* Endogenous hepadnavirus, *ECV* Endogenous circoviral element, *HERV* Human endogenous retrovirus, *muERV* Murine endogenous retrovirus, *AVP* NCBI all virus proteins set

^a^DIGS was used as part of “phylogenetic screening” pipeline (see Fig. 1b)

^b^DIGS-based investigations were allied to experimental or functional genomics investigations

^c^Indicates subset of the RSL from which probes were derived (note that Retroviridae here denotes both endogenous and exogenous retroviruses)

While straightforward in principle*,* in silico genome screening is computationally expensive and can be difficult to implement efficiently. Moreover, large-scale screens can produce copious output data that are difficult to manage and interpret without an appropriate analytical framework. To address these issues, we developed a database-oriented approach to in silico screening, called *database-integrated genome screening* (DIGS). To demonstrate the use of this approach, we first created an open software framework for performing it, then used this framework to search published vertebrate genomes for EVE loci. Besides demonstrating that DIGS provides a powerful, flexible approach for exploring the dark genome, our analysis provides a comprehensive and detailed overview of EVE diversity in vertebrate genomes and reveals new information about the long-term evolutionary relationships between viruses and vertebrate hosts.

## Results

### A database-integrated approach to exploring the dark genome

We developed a robust, database-integrated approach to systematic in silico genome screening, referred to as database-integrated genome screening (DIGS). This approach integrates a similarity search-based screening pipeline with a relational database management system (RDBMS) to enable efficient exploration of the dark genome. The rationale for this integration is twofold: it not only provides a solid foundation for conducting large-scale, automated screens in an efficient and non-redundant manner but also allows for the structured querying of screening output using SQL, a powerful and well-established tool for database interrogation [41]. Additionally, an RDBMS offers advantages such as data recoverability, multi-user support, and networked data access.

The DIGS process comprises three key input data components:

- Target database (TDb): A collection of whole genome sequence assemblies (or other large sequence datasets such as transcriptomes) that will serve as the target for sequence similarity searches.
- Query sequences (Probes): A set of sequences to be used as input for similarity searches of the TDb.
- Reference sequence library (RSL): The RSL represents the broad range of genetic diversity associated with the genome feature(s) under investigation. Its composition varies according to the analysis context (see Table 1). It should always include sequences representing diversity within the genome feature under investigation. It may also include genetic marker sequences and potentially cross-matching genome features. Probes are typically a subset of sequences contained in the RSL.

**Fig. 2:**
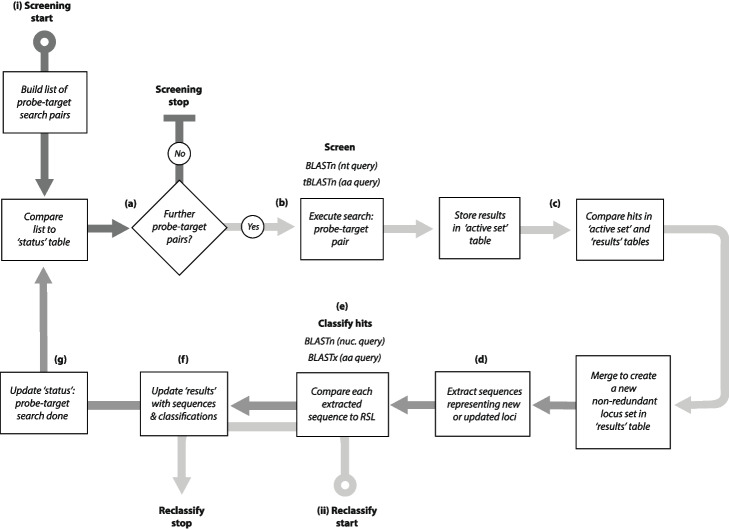
The database-integrated genome screening (DIGS) process as implemented in the DIGS tool. (i) Screening. **a** On initiation of screening a list of searches, composed of each query sequence versus each target database (TDb) file is composed based on the probe and TDb paths supplied to the DIGS program. Subsequently, screening proceeds systematically as follows: **b** the status table of the project-associated screening database is queried to determine which searches have yet to be performed. if there are no outstanding searches then screening ends, otherwise it proceeds to step **b** wherein the next outstanding search of the TDb is performed using the selected probe and the appropriate BLAST+ program. Results are recorded in the data processing table (“active set”); **c** results in the processing table are compared to those (if any) obtained previously to derive a non-redundant set of non-overlapping loci, and an updated set of non-redundant hits is created, with each hit being represented by a single results table row. To create this non-redundant set, hits that overlap, or occur within a given range of one another, are merged to create a single entry. **d** Nucleotide sequences associated with results table rows are extracted from TDb files and stored in the results table; **e** extracted sequences are classified via BLAST-based comparison to the RSL using the appropriate BLAST program. **f** The header-encoded details of the best-matching sequence (species name, gene name) are recorded in the results table. **g** The status table is updated to create a record of the search having been performed, and the next round of screening is initiated. (ii) Reclassification: hits in the results table can be reclassified following an update to the reference sequence library

As illustrated in Fig. 2, the DIGS process involves systematic searching of a user-defined TDb with user-defined probes, merging fragmented hits, and classifying merged sequences through BLAST-based comparison to the RSL. The output—a set of non-redundant, defragmented hits—is captured in a project-specific relational database. Importantly, this integration allows database queries to be employed in real time, with SQL queries referencing any information captured by the database schema. SQL-based querying of screening databases facilitates the identification of loci of interest, which can then be explored further using comparative approaches (see Fig. 1b).

It is important to note that screening is usually an iterative discovery process, wherein initial results inform the development of subsequent screens. For instance, novel diversity detected by an initial screen can subsequently be incorporated into the RSL and hits within the screening database can be reclassified using the updated library (Fig. 2). Additionally, probe sets used in initial searches can be expanded to incorporate sequences identified during screening, broadening the range of sequences detected in subsequent screens [42]. However, care must be taken when using this approach, since it can potentially produce misleading results, or generate excessive hits (e.g., if highly repetitive sequences are contained within the new probes). Database integration allows screening results to be observed and interrogated in real time—as they are being generated. This means that configuration issues (e.g., badly composed RSL, inappropriate choice of probes) can be detected early on—potentially saving a significant amount of time and effort. Furthermore, it facilitates the implementation of agile, heuristic screening strategies, in which approaches are adjusted in line with results.

### An open software framework for implementing DIGS

We constructed a software framework for implementing DIGS, called “the DIGS tool”. The DIGS tool is implemented using the PERL scripting language. It uses the BLAST + program suite [24] to perform similarity searches and the MySQL RDBMS (to capture their output). Accessible through a text-based console interface, it simplifies the complex process of large-scale genome screening and provides a versatile basis for implementing screens.

To initiate screening using the DIGS tool, researchers provide a project-specific command file (Additional file 1: Fig. S1) that serves as the blueprint for the screening process. This command file specifies parameters for BLAST searches, the user-defined name of the screening database, and file paths to the TDb, RSL, and probe sequences. When a screen is initiated, a project-specific database is created. This core schema (Additional file 2: Fig. S2) can subsequently be extended to include any relevant “side data”—e.g., taxonomic information related to the species and sequences included in the screen—increasing the power of SQL queries to reveal informative patterns (Additional file 3: Fig. S3).

Systematic screening proceeds automatically until all searches have been completed. If the process is interrupted at any point, or if novel probe/target sequences are incorporated into the project, screening will proceed in a non-redundant way on restarting. Thus, screening projects can expand as required to incorporate new TDb files (e.g., recently published WGS assemblies) or novel probe/reference sequences. The DIGS tool console allows reclassification of sequences held in the results table (e.g., following an RSL update). To increase efficiency, this process can be tailored to specific subsets of database sequences by supplying SQL constraints via the DIGS tool console.

BLAST algorithms emphasize local similarity and consequently tend to fragment contiguous matches into several separate hits if similarity across some internal regions of the match is low. The DIGS tool allows screening pipelines to be configured with respect to how overlapping/adjacent hits are handled, so that the screening process can be tailored to the specific needs of diverse projects. The DIGS tool also provides a “consolidation” function that concatenates, rather than merges, adjacent hits and stores concatenated results, along with information about their structure, in a new screening database table.

For program validation, we mined mammalian genomes for sequences disclosing similarity to the antiviral restriction factor tetherin [43, 44]. Tetherin provides a useful test case as it is a relatively distinctive gene and its evolution, distribution and diversity have previously been examined [43, 44]. Results were compared with those provided by two alternative, widely used genome mining pipelines: OrthoDB [45] and Ensembl [46] and found to overlap by > 99% (Additional file 4: Fig. S4).

The DIGS tool provides functionality for exporting FASTA-formatted sequences and managing screening database tables (e.g., add/drop tables, import table data). Further information regarding program installation and usage is provided online, in a repository associated website [47]. In the sections below, we illustrate the application of the DIGS tool to cataloging of EVEs in vertebrate genomes, focussing on both high and low copy number elements.

### Use of DIGS to catalog RT-encoding endogenous retroviruses

Unusually among vertebrate viruses, retroviruses (family *Retroviridae*) integrate their genome into the nuclear genome of infected cells as an obligate part of their life cycle. As a result, retroviruses gain more opportunities to become a permanent part of the host germline. Furthermore, the initial integrated form of a retrovirus genome, called a provirus, is typically replication competent. ERVs can therefore increase their germline copy number through reinfection of germ line cells or (after adaptation) by intracellular retrotransposition [48, 49]. Accordingly, “endogenous retroviruses” (ERVs) are by far the most common type of EVE found in vertebrate genomes [7, 50].

Retrovirus genomes contain a *pol* coding domain that encodes a reverse transcriptase (RT) gene. The RT gene can be used to reconstruct phylogenetic relationships across the entire Retroviridae and hence provides the lynchpin for unraveling the evolutionary history and origins of ERV loci [51, 52]. We therefore implemented a screening procedure to detect RT-encoding ERV loci, based on an RSL comprised of previously classified exogenous retrovirus and ERV RT sequences (see “Materials and methods”). Screening involved more than 1.5 million discrete tBLASTn searches and resulted in the identification of 1,073,137 ERV RT hits. This set was filtered based on higher BLAST bitscore cutoff to obtain a high confidence set of 702,167 loci (Table 2).

**Table 2:** ERV RT loci identified via in silico screening

**Vertebrate class**	**# WGS**	**Retrovirus clade**					
		**Clade I**		**Clade II**		**Clade III**	
		**Total #**	**Average #**	**Total #**	**Average #**	**Total #**	**Average #**
Agnatha	3	32	*10.67*	1^a^	*0.33*	300	*100.00*
Chondrichthyes	6	2018	*336.33*	0	*0.00*	2843	*473.83*
Actinopterygii	173	8514	*49.21*	64^a^	*0.37*	2177	*12.58*
Actinistia	1	0	*0.00*	0	*0.00*	97	*97.00*
Amphibia	34	17,319	*509.38*	973	*28.62*	8019	*235.85*
Reptilia	92	13,676	*148.65*	12,120	*131.74*	20,197	*219.53*
Aves	143	17,951	*125.53*	20,797	*145.43*	42,014	*293.80*
Mammalia	452	215,304	*476.34*	174,549	*386.17*	143,364	*317.18*

*WGS* Whole genome sequence assemblies screened

^a^Hits likely due to contamination

**Fig. 3:**
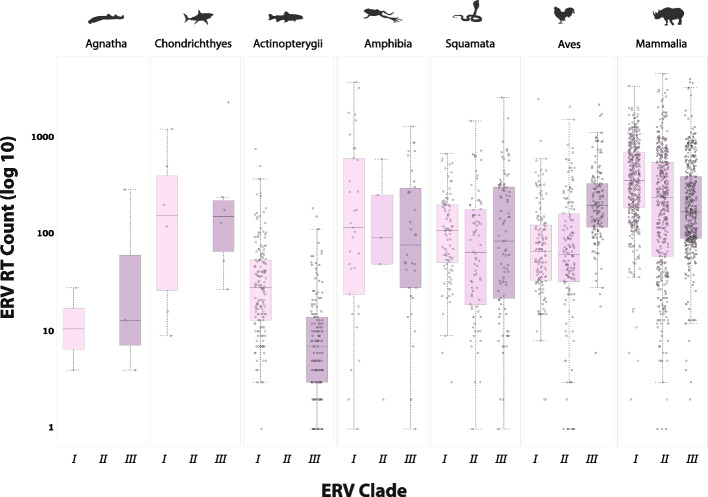
Counts of ERV RT loci identified by identified via database integrated genome screening of 874 vertebrate species. Box plots show the distribution of endogenous retrovirus (ERV) reverse transcriptase (RT) counts in distinct vertebrate classes. Median and range of values are indicated. Circles indicate counts for individual species. Counts are shown against a log scale. Figure created in R using ggplot2 and geom_boxplot. RT hits identified as likely contaminants are not shown

High confidence ERV RT hits were identified in all vertebrate classes. However, the frequency among classes was found to vary dramatically (Fig. 3). ERVs occur most frequently in mammals (class Mammalia) and amphibians (class Amphibia), and at relatively similar, intermediate frequencies in the genomes of reptiles (class Squamata) and birds (class Aves). By contrast, RT-encoding ERVs are relatively rare in the genomes of fish, including ray-finned fish (class Actinopterygii) and jawless fish (class Agnatha). Cartilaginous fish (class Chondrichthyes) represent a possible exception, although only a few genomes were available for this group (Fig. 3). These findings are broadly consistent with previous studies, conducted using a smaller number of species genomes [50, 53–55].

ERVs have been taxonomically grouped into three clades (I, II, and III) based on their phylogenetic relatedness in the RT gene to the exogenous *Gammaretrovirus*, *Betaretrovirus*, and *Spumavirus* genera, respectively [1, 2]. We incorporated into our RT screening database taxonomic information for (i) host species examined in our screen and (ii) RSL RT sequences. We then used an SQL query referencing these tables to summarize the frequency of clade I, II and III ERVs in distinct vertebrate classes (Additional file 3: Fig. S3). Whereas clade I and III ERVs are found in all vertebrate groups, clade II ERVs appear to have a more restricted distribution, occurring only at low frequency in amphibians, and being completely absent from agnathans and cartilaginous fish (Table 2). A few clade II ERVs were identified in ray-finned fish, but these were very closely related to mammalian ERVs and likely represent contamination of WGS builds with mammalian genomic DNA. While RT-encoding ERV copy number is quite high in cartilaginous fish, RT diversity is relatively low, with the majority of ERV RT sequences belonging to clade III.

### Use of DIGS to catalog non-retroviral EVEs vertebrate genomes

To identify non-retroviral EVEs, we first obtained an RSL representing all known viruses [56]. From this library, a set of representative probes was selected. Probes comprised representative proteomes of all known vertebrate viruses except retroviruses. Screening entailed > 1.5 million discrete tBLASTn searches, and initial results comprised 33,654 hits. However, many of these represented matches to host genes and TEs. We identified these spurious matches by interrogating screening results with a combination of SQL queries, BLAST-based comparisons to curated sequence databases, and ad hoc phylogenetic analysis.

We excluded hits that contained intact coding regions and lacked evidence of integration into host DNA, since these may be derived from contaminating exogenous viruses (Additional file 5: Table S1). We also excluded other virus-derived DNA sequences that appeared likely to represent diet-related contamination of WGS data. For example, SQL-generated summaries of our initial screen results revealed several sequences disclosing similarity plant viruses, including geminiviruses (family *Geminiviridae*) and potyviruses (family *Potyviridae*) (Additional file 3: Fig. S3). These sequences contained multiple stop codons and frameshifts, suggesting they might represent EVEs embedded within contaminating DNA, particularly since EVEs derived from both these virus groups are known occur in plant genomes [57, 58]. Other unexpected matches to plant virus groups were contained within large contigs and thus could not be dismissed as contaminating DNA. For example, a sequence identified in the genome of the pig-nosed turtle (*Carettochelys insculpta*) disclosed similarity to caulimoviruses (family *Caulimoviridae*). However, genomic analysis revealed this sequence in fact represents an unusual ERV (Additional file 6: Fig. S5).

**Fig. 4:**
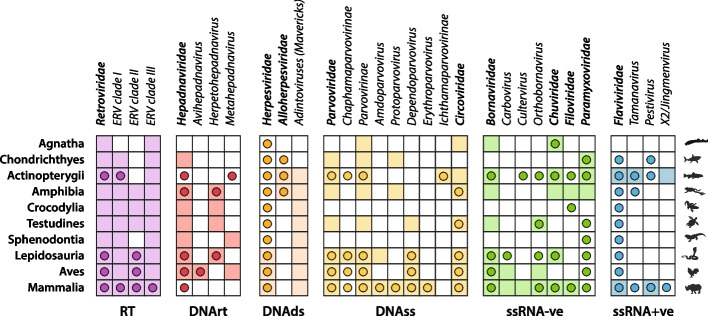
Exogenous versus endogenous distribution of virus families that have been incorporated into the vertebrate germline. Circles indicate the known presence of exogenous viruses in vertebrate groups, determined through reference to the NCBI virus genomes resource [56], supplemented with information obtained from recently published papers [64–70]. Shaded boxes indicate the presence of endogenous viral elements, as determined in the present study. RT retroviruses, DNArt reverse transcribing DNA viruses, DNAss single-stranded DNA viruses, DNAds double-stranded DNA viruses, RNAds double-stranded RNA viruses, RNAss-ve single-stranded negative sense RNA viruses, RNAss + ve single-stranded positive sense RNA viruses

Next we removed matches to recognized transposons that are wholly or partly comprised of virus-derived DNA, such as polintons/mavericks [59–61] and teratorns [62] (Additional file 3: Fig. S3). Once these EVE-like TEs had been removed, results comprised 6038 putative non-retroviral EVE sequences, representing 10 virus families (Table 3, [63]). We did not identify any EVEs derived from vertebrate viruses with genomes comprised of double-stranded RNA (e.g., order Reovirales) or circular single-stranded RNA (e.g., genus *Deltavirus*). However, all other virus genome “classes” were represented including reverse-transcribing DNA (DNArt) viruses, double-stranded DNA (DNAds) viruses, single-stranded DNA (DNAss) viruses, single-stranded negative sense RNA (RNAss-ve) viruses, and single-stranded positive sense RNA (RNAss + ve) viruses. Plotting the distribution of EVEs and exogenous viruses from distinct virus families and genera across vertebrate phyla revealed that many virus groups have had a broader distribution across vertebrate hosts than recognized on the basis of previously identified exogenous viruses (Fig. 4).

**Table 3:** Number of non-retroviral EVE sequence identified and estimated number of germline incorporation events in distinct vertebrate classes

**Virus group**	**# EVEs identified (estimated # germline incorporation events)**															
	**Total**		**Mammalia**		**Aves**		**Reptilia**		**Amphibia**		**Actinopterygii**		**Chondrichthyes**		**Agnatha**	
**ssRNA-ve**																
Bornaviridae	2566	(383)	2434	(292)	30	(11)	27	(14)	52	(44)	22	(21)	-	-	1	(1)
Chuviridae	182	(164)	24	(24)	-	-	23	(23)	9	(9)	119	(108)	-	-	7	-
Filoviridae	390	(69)	389	(68)	-	-	-	-	1	(1)	-	-	-	-	-	-
Paramyxoviridae	19	(17)	-	-	-	-	-	-	4	(3)	14	(3)	1	(1)	-	-
**ssRNA + ve**																
Flaviviridae	8	(11)	1	(1)	-	-	-	-	-	-	7	(10)	-	-	-	-
**DNArt**																
Hepadnaviridae	993	(108)	-	-	897	(89)	93	(17)	2	(1)	-	-	1	(1)	-	-
**DNAss**																
Circoviridae	1198	(131)	918	(29)	32	(15)	91	(19)	82	(24)	68	(38)	-	-	7	(6)
Parvoviridae	689	(238)	534	(199)	34	(10)	34	(13)	12	(7)	12	(6)	3	(3)	-	-
**DNAds**																
Herpesviridae	13	(8)	11	(6)	1	(1)	1	(1)	-	-	-	-	-	-	-	-
Alloherpesviridae	28	(8)	-	-	-	-	-	-	15	(3)	13	(5)	-	-	-	-
**Total**	6087	(1137)	4311	(619)	994	(126)	269	(87)	177	(92)	255	(191)	5	(5)	15	(7)

Germline incorporation here implies both integration and fixation in the germline

We examined all EVE loci identified in our study to determine their coding potential. We identified numerous EVE loci encoding open reading frames (ORFs) > 300 amino acids (aa) in length (Additional file 7: Fig. S6). Among these, 4 encoded ORFs longer than 1000 aa. One of these—a 1718aa ORF encoded by an endogenous borna-like L-protein (EBLL) element in bats (EBLL-Cultervirus.29-EptFus) —has been reported previously [71]. However, we also identified an endogenous chuvirus-like L-protein (ECLL) element encoding an ~ 1400 aa ORF in livebearers (subfamily Poeciliinae). This element encodes long ORFs in two distinct livebearer species (*P. formosa and P. latapina*), indicating its coding capacity has been conserved for > 10 million years [72]. We also detected herpesvirus and alloherpesvirus EVEs encoding ORFs > 1000 aa, but as discussed below, the integration status of these sequences remains unclear.

### Diversity of non-retroviral EVEs in vertebrate genomes

#### EVEs derived from viruses with double-stranded DNA genomes

We detected DNA derived from herpesviruses (family *Herpesviridae*) in mammalian and reptilian genomes (Fig. 4, Table 3, [63]). DNA sequences derived from betaherpesviruses (subfamily *Betaherpesvirinae*) and gammaherpesviruses (subfamily *Gammaherpesvirinae*) have previously been reported in WGS assemblies of the tarsier (*Carlito syrichta*) and aye-aye (*Daubentonia madagascensis*), respectively [73]. In addition to these sequences, we detected gammaherpesvirus DNA in WGS data of red squirrels (*Sciurus vulgaris*) and the Amazon river dolphin (*Inia geoffrensis*), while betaherpesvirus DNA was detected in the stoat (*Mustela ermina*) WGS assembly, and DNA derived from an alphaherpesvirus (subfamily *Alphaherpesvirinae*) in the Oriximina lizard (*Tretioscincus oriximinensis*) WGS (Additional file 8: Fig. S7). Germline integration of human betaherpesviruses has been demonstrated [74, 75], and the presence of a betaherpesvirus-derived EVE in the tarsier genome EVE has been established [73]. However, herpesviruses can also establish latent infections and might be considered likely to occur as contaminants of DNA samples used to generate whole genome sequence assemblies. Due to the limitations of the WGS assemblies in which they were identified, it was not possible to confirm that the novel herpesvirus DNA sequences detected here represent EVEs rather than DNA derived from contaminating exogenous viruses.

DNA derived from alloherpesviruses (family *Alloherpesviridae*) was detected in fish and amphibians. In ray-finned fish, most of these sequences belonged to the “teratorn” lineage of transposable elements, which have arisen via fusion of alloherpesvirus genomes and piggyBac transposons, and have been intragenomically amplified in the genomes of teleost fish (infraclass Teleostei) [62]. Additional alloherpesvirus-related elements were identified in three amphibian species and five ray-finned fish species [63]. One of these elements, identified in the Asiatic toad (*Bufo gargarizans*) occurred within a contig that was significantly larger than a herpesvirus genome, demonstrating that it represents an EVE rather than an exogenous virus. Phylogenetic analysis revealed that alloherpesvirus-like sequences identified in amphibian genomes clustered robustly with amphibian alloherpesviruses, while those identified in fish genomes clustered with fish alloherpesviruses (Additional file 8: Fig. S7).

#### EVEs derived from viruses with single-stranded DNA genomes

EVEs derived from parvoviruses (family *Parvoviridae*) and circoviruses (family *Circoviridae*) are widespread in vertebrate genomes, being found in the majority of vertebrate classes (Fig. 4). Both endogenous circoviral elements (ECVs) and endogenous parvoviral elements (EPVs) are only absent in major vertebrate groups represented by a relatively small number of sequenced species genomes (i.e., between 1 and 6). No ECVs or EPVs were identified in the tuatara (order Rhynchocephalia) or in crocodiles (order Crocodilia). EPVs were not identified in agnathans, while ECVs were not identified in cartilaginous fish.

We identified a total of 1192 ECVs, most of which are derived from elements in carnivore (class Mammalia: order Carnivora) genomes that are embedded within non-LTR retrotransposons and have undergone intragenomic amplification (Additional file 9: Fig. S8). While many of the ECVs identified in our screen have been reported in previous publications [7, 32, 36, 42, 76], we also identified novel loci in mammals, reptiles, amphibians, and ray-finned fish [63]. Phylogenetic analysis (see Additional file 8: Fig. S7) revealed that a novel ECV locus in turtles groups with avian circoviruses, while amphibian ECV elements grouped with fish circoviruses, though bootstrap support for this relationship was lacking. A circovirus-like sequence detected in the WGS data of Allen’s wood mouse (*Hylomyscus alleni*) grouped robustly with exogenous rodent circoviruses, but integration of this sequence into the *H. alleni* genome could not be confirmed.

We identified 627 EPVs, representing two distinct subfamilies within the Parvoviridae and five distinct genera (see Fig. 4). The majority of these loci have been reported in a previous study [32] or are orthologs of these loci. However, we identified novel EPVs in reptiles, amphibians and mammals (Table 3, [63]). In reptiles the novel elements derived from genus *Dependoparvovirus* while the amphibian elements were more closely related to viruses in genus *Protoparvovirus*. Notably, the novel amphibian EPVs clustered basally within a clade of protoparvovirus-related viruses in phylogenetic reconstructions (Additional file 8: Fig. S7), consistent with previous analyses indicating that protoparvovirus ancestors may have broadly co-diverged with vertebrate phyla [32].

#### EVEs derived from reverse-transcribing DNA viruses

EVEs derived from hepadnaviruses (family *Hepadnaviridae*), which are reverse-transcribing DNA viruses, were identified in reptiles, birds and amphibians (Table 3, [63]). Most of these EVEs, commonly referred to as “endogenous hepatitis B viruses” (eHBVs), have been reported previously [35, 77]. However, we identified novel elements in the plateau fence lizard (*Sceloporus tristichus*) and others in vertebrate classes where eHBVs have not been reported previously. These include one element identified in a cartilaginous fish, the Australian ghostshark (*Callorhinchus milii*), and another identified in an amphibian, the common coquí (*Eleutherodactylus coqui*).

Phylogenetic analysis (see Additional file 8: Fig. S7) revealed that novel eHBV elements identified in lizards (suborder Lacertilia) group robustly with the exogenous skink hepadnavirus (SkHBV), while the amphibian element groups within a clade comprised of the exogenous spiny lizard hepadnavirus (SlHBV), Tibetan frog hepadnavirus (TfHBV) and eHBV elements identified in crocodile genomes. The eHBV identified in sharks was relatively short and not amenable to phylogenetic analysis but nonetheless provides the first evidence that hepadnaviruses infect this host group.

#### EVEs derived from viruses with single-stranded, negative sense RNA genomes

Screening revealed that vertebrate genomes contain numerous EVEs derived from mononegaviruses (order *Mononegavirales*), which are characterized by non-segmented ssRNA-ve genomes. These EVEs derive from four mononegavirus families: bornaviruses (family *Bornaviridae*), filoviruses (family *Filoviridae*), paramxyoviruses (family *Paramyxoviridae*) and chuviruses (family *Chuviridae*) (Fig. 4, Table 3, [63]). We did not detect any EVEs derived from other mononegavirus families that infect vertebrates (*Pneumoviridae*, *Rhabdoviridae*, *Nyamiviridae*, *Sunviridae*), nor any EVEs derived from virus families with segmented, negative sense RNA genomes (e.g., *Peribunyaviridae*, *Orthomyxoviridae*).

The majority of mononegavirus EVEs identified in our screen were derived from bornaviruses and filoviruses and have been described in previous reports [7, 32, 35, 36, 78]. However, we also identified novel EVEs derived from these groups, as well as previously unreported EVEs derived from paramyxoviruses and chuviruses (Table 3).

Germline integration of DNA derived from mononegaviruses can occur if, in an infected germline cell, viral mRNA sequences are reverse transcribed and integrated into the nuclear genome by cellular retroelements [79]. EVE loci generated in this way preserve the sequences of individual genes of ancient mononegaviruses, but not entire viral genomes. Among mononegavirus-derived EVEs, regardless of which family, elements derived from the nucleoprotein (NP) and large polymerase (L) genes predominate. However, other genes are also represented, including the glycoprotein (GP) genes of filoviruses, bornaviruses, and chuviruses, the VP30 and VP35 genes of filoviruses, and the hemagglutinin-neuraminidase (HA-NM) gene of paramyxoviruses.

Paramyxovirus-like EVEs were identified in ray-finned fish, amphibians, and sharks (Fig. 4, Table 3, [63]). Many of these EVEs were highly divergent and/or degenerated and consequently their evolutionary relationships to contemporary paramyxoviruses were poorly resolved in phylogenetic analysis. However, an L polymerase-derived sequence identified in the pobblebonk frog (*Limnodynastes dumerilii*) genome was found to group robustly with Sunshine Coast virus, a contemporary paramyxovirus of Australian pythons [80] in phylogenetic trees (Additional file 8: Fig. S7).

Chuvirus-like sequences were identified in agnathans, ray-finned fish, reptiles, amphibians, and mammals (Fig. 4, Table 3, [63]). The majority of the mammalian elements were identified in marsupials, but we also identified a single chuvirus-like EVE in the genome of a laurasiatherian mammal—the bottlenose dolphin (*Tursiops truncatus*). Phylogenetic trees reconstructed using alignments of NP-derived chuvirus EVEs and NP genes of contemporary chuviruses revealed evidence for the existence of distinct clades specific to particular vertebrate classes (Additional file 8: Fig. S7). These included a clade including both a snake EVE and an exogenous chuviruses of snakes, and two clades comprised of EVEs and viruses of teleost fish. In addition, these phylogenies revealed a robustly supported relationship between chuvirus EVEs in the Tibetan frog (*Nanorana parkeri*) and zebrafish (*Danio rerio*) genomes. Taken together, these results provide evidence for the existence of numerous diverse lineages of chuviruses in vertebrates, adding to recent evidence for the presence of exogenous chuviruses in marsupials [64].

Filovirus-derived EVEs were mainly identified in mammals (Fig. 4, Table 3, [63]). However, we also identified one filovirus-derived EVE in an amphibian—the mimic poison frog (*Ranitomeya imitator*) —providing the first evidence that filoviruses infect this vertebrate group (Table 1). Among mammals, we identified novel, ancient filovirus EVEs in anteaters (family Myrmecophagidae) and spiny mice (genus *Acomys*).

**Fig. 5:**
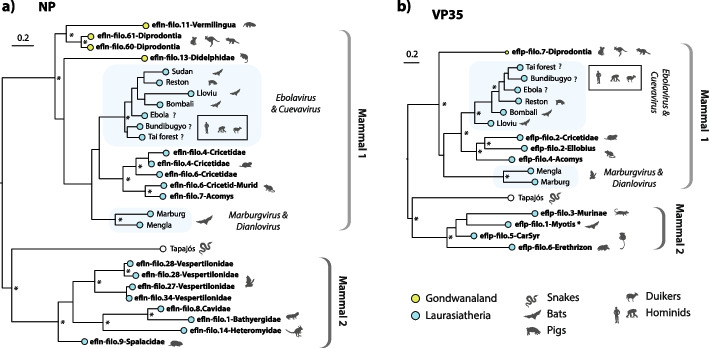
Evolutionary relationships of filoviruses and filovirus-derived EVEs. Bootstrapped maximum likelihood phylogenies showing the evolutionary relationships between filoviruses and filovirus EVEs in the nucleoprotein (NP) and viral protein 35 (VP35) genes. Phylogenies were constructed using maximum likelihood as implemented in RAxML, and codon-aligned nucleotides for each gene. Numbers adjacent internal nodes indicate bootstrap support (1000 bootstrap replicates). The scale bar indicates evolutionary distance in substitutions per site. Virus taxon names are shown in regular font, EVE names are shown bold. EVE names follow standardized nomenclature (see “Materials and methods”). Brackets to the right of each tree indicate virus genera (italics) and major lineages (bold). Silhouettes indicate host groups following the key. For Ebola virus, Bundibugyo virus, and Tai Forest virus, the main reservoir hosts are unknown. The inset box adjacent these taxa show host species in which one or more of these viruses has been isolated [81, 82], following the key. *Experimentally investigated locus [83, 84]

Strikingly, the inclusion of Tapajos virus (TAPV), a snake filovirus, in phylogenetic reconstructions revealed evidence for the existence of two highly distinct filovirus lineages in mammals (Fig. 5). These two lineages, which are robustly separated from one another by TAPV, are evident in phylogenies constructed for both the NP and VP35 genes. One lineage (here labeled “Mammal-1”) is comprised of EVEs and all contemporary mammalian filoviruses, whereas the other (“Mammal-2”) is comprised exclusively of EVEs. Notably, within the Mammal-1 group, EVEs identified in host species groups that are indigenous to Southern Hemisphere continents (e.g., marsupials, xenarthrans) cluster basally, whereas EVEs and viruses isolated from “Old World”-associated placental mammals occupy a more derived position.

The “Mammal-2” clade contains filovirus EVEs from rodents, primates, and bats. Because EVEs belonging to this clade were obtained from several distinct lineages, and show conservation across these groups, we can be reasonably confident they represent a *bona fide* lineage within the Filoviridae, rather than just a set of highly degraded filo-like EVEs that group together due to long branch attraction [85]. One member of this group (eflp-filo.1-Myotis) encodes an intact VP35 protein, the properties of which have been experimentally investigated in recent studies [83, 84]. Interestingly, we found that spiny mice also harbor a filovirus EVE encoding an intact VP35 protein (eflp-filo.3-Acomys); however, this insertion belongs to the “Mammal 1” clade and is relatively closely related to the VP35 proteins found in contemporary mammalian filoviruses (Fig. 5b).

Bornavirus-like EVEs were identified in all vertebrate classes except Chondrichthyes (Fig. 4, Table 3, [63]). The majority have been reported previously or are orthologs of previously reported EVEs. However, we identified novel bornavirus-like EVEs in the genomes of ray-finned fish and amphibians. The amphibian EVEs grouped robustly with culterviruses in phylogenetic reconstructions (Additional file 8: Fig. S7).

#### EVEs derived from viruses with single-stranded, positive sense RNA genomes

EVEs derived from positive sense RNA viruses are rare in vertebrate genomes (Fig. 4, Table 3, [63]). The only examples we identified were a small number of sequences derived from flavivirids (family *Flaviviridae*). These include an EVE derived from the *Pestivirus* genus of flavivirids, the reference genome of the Indochinese shrew (*Crocidura indochinensis*), as reported previously [86], and EVEs identified in ray-finned fish, also reported previously [31]. In fish genomes, flavivirid EVEs derive from the proposed “Tamanavirus” genus, and a lineage labeled “X2” that groups as a sister taxon to the proposed “Jingmenvirus” genus. However, jingmenviruses are actually segmented, RNAss-ve viruses whose genomes include flavivirid-derived segments [87]. Since it is possible that the X2 lineage shares a common RNAss-ve ancestor with jingmenviruses, EVEs belonging to this lineage may in fact be derived from viruses with ssRNA-ve genomes.

#### Frequency of germline incorporation events across distinct vertebrate phyla

We used the DIGS framework to dissect the history of horizontal gene transfer events involving germline incorporation of DNA derived from non-retroviral viruses. We excluded EVEs derived from Polinton-like viruses (Adintoviruses) and teratorn elements, both of which exhibit relatively high copy numbers due to intragenomic amplification [60–62, 88]. For these groups, the large number of insertions, and the fact that amplified lineages appear to have been independently established on multiple occasions, meant that such an analysis would be beyond the scope of this study.

To examine the rate of germline incorporation in the remaining groups of non-retroviral EVEs, we compiled an expanded RSL containing a single reference sequence for each putative (or previously confirmed) ortholog. By classifying our hits against this expanded RSL, we could discriminate novel EVE loci (paralogs) from orthologs of previously described EVE loci. Where novel paralogs were identified, we incorporated these into our RSL and then reclassified related sequences in our screening database against this updated library. By investigating loci in this way, and iteratively reclassifying database sequences, we progressively resolved the various non-retroviral EVEs identified in our screen into sets of putatively orthologous insertions. Via this analysis, we estimated that the non-retroviral EVEs identified in our study (excluding those derived from DNAds viruses) represent ~1137 distinct germline incorporation events (Table 3). Using orthology information, we calculated minimum age estimates for all non-retroviral EVEs identified in two or more species [63]. We applied standardized nomenclature to EVE loci (see “Materials and methods”), capturing information about EVE orthology, taxonomy and host distribution [63].

**Fig. 6:**
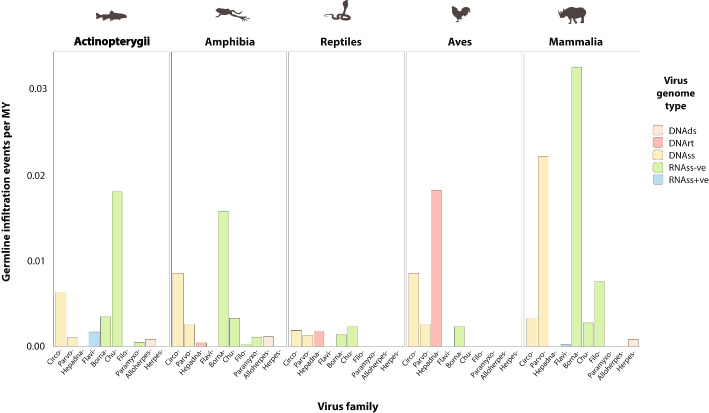
Comparison of germline infiltration rates in five vertebrate classes. Infiltration rates represent the rate of incorporation and fixation per million years (MY) of species branch length sampled. Rates are shown for each non-retroviral family represented by vertebrate EVEs. Colors indicate reverse transcribing DNA (DNArt) viruses, single-stranded DNA (DNAss) viruses, single-stranded negative sense RNA (RNAss-ve) viruses, and single-stranded positive sense RNA (RNAss-ve) viruses, following the key

Next, we estimated the rate of germline incorporation for each endogenized virus family, in all vertebrate classes represented by at least ten species (Fig. 6). Rates were found to vary dramatically across each of the vertebrate groups examined. Overall, rates were highest in mammals and lowest in reptiles. Fish and amphibians disclosed similar rates with DNAss and ssRNA-ve viruses being incorporated at similar, intermediate rates. Birds were generally similar to reptiles but show a higher rate of DNAss virus incorporations and a markedly elevated rate of hepadnavirus incorporation. Rates of parvovirus, filovirus, and bornavirus infiltration were very high in mammals compared to other vertebrate classes, with bornaviruses being incorporated at a particularly high rate (> 0.03 per million years of species evolution). A relatively high rate of incorporation of RNAss + ve viruses was observed in ray-finned fish, but since the elements in question are closely related to jingmenviruses, as described above, they may in fact reflect incorporation of DNA derived from an RNAss-ve virus group [87].

**Fig. 7:**
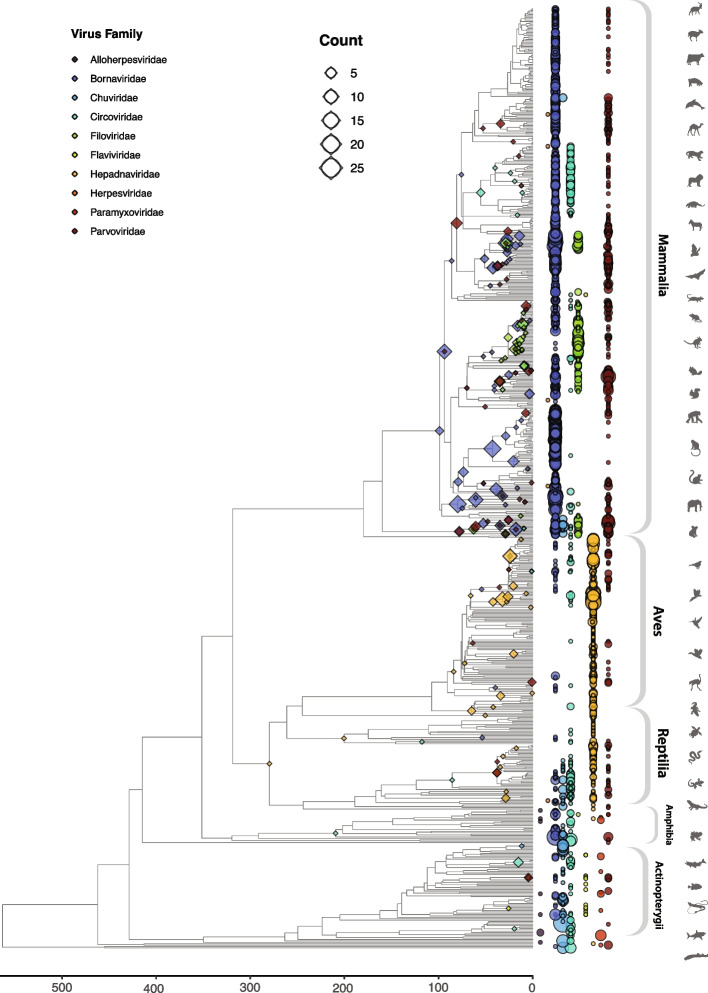
Overview of germline incorporation in vertebrates. A time-calibrated phylogeny of vertebrate species examined in this study, obtained via TimeTree [89]. Minimum ages of endogenization events are indicated by diamonds on internal nodes for EVE loci present as orthologs in multiple species. The presence of EVE sequences in each species genome is indicated by circles at phylogeny tips. Circles and diamonds nodes are scaled by the number of sequences detected and color-coded by virus family as indicated in legend. For circles, scaling indicates the total number of EVE sequences detected within each species genome, including both unique and shared endogenization events

In addition to estimating the frequency of germline incorporation of non-retroviral viruses, we used our screening data to reconstruct a time-calibrated overview of virus integration throughout vertebrate evolutionary history (Fig. 7, Additional file 10: Table S2, Additional file 11: Fig S9). Among putatively orthologous groups of EVEs for which we were able to estimate minimum dates of integration, the majority were found to have been incorporated in the Cenozoic Era (1-66 Mya). So far, the oldest integration event identified involves a metahepadnavirus (genus *Metahepadnavirus*)-derived EVE that appears to be orthologous in tuataras and birds, indicating it was incorporated into the saurian germline > 280–300 Mya (see [35]). Other ancient EVEs include circovirus and herpetohepadnavirus (genus *Herpetohepadnavirus*)-derived EVEs in turtles (order Testudines) (see [77]), a circovirus-derived EVE in frogs (order Anura), and bornavirus integrations in placental mammals (see [78]). Besides revealing the landscape of non-retroviral EVE integration throughout vertebrate history, plotting EVE distribution in this way clearly reveals the main differences in EVE distribution across host groups (Fig. 7).

## Discussion

Sequencing of genomes is advancing rapidly but deciphering the complex layers of information they contain is a challenging, long-term endeavor [78, 79]. Genomes are not only inherently complex but they also exhibit remarkable dynamism, with phenomena such as recombination, transposition, and horizontal gene transfer contributing to the creation of genomic “churn” that makes feature distribution difficult to map [80]. These issues, combined with rapid data accumulation, coverage limitations, and assembly errors—make generation of complete and accurate annotations difficult [83, 85]. Consequently, labor-intensive manual genome annotation remains important [64, 78], and most published whole genome sequences are comprised of genomic “dark matter”.

An exciting aspect of these circumstances is that they provide immense scope to make interesting biological discoveries using low cost, approaches. While experimental studies are generally required to characterize genome features at a functional level, approaches based solely on comparative sequence analysis (see Fig. 1b) can often reveal useful insights into their biology and evolution [1, 90]. Furthermore, comparative investigations in silico can often be productively combined with functional genomics or experimental approaches (Fig. 1b, Table 1).

Systematic in silico genome screening is computational approach that facilitates investigation of the dark genome (Fig. 1). However, it can be challenging to implement efficiently. Automated pipelines are generally required to implement large-scale screens [91], and these can produce copious output data that are difficult to manage and interpret without an appropriate analytical framework. Here, we introduce DIGS—a robust analytical platform for conducting large-scale in silico screens—and describe an open software framework (the DIGS tool) for implementing it.

EVEs constitute one interesting and informative group of genome features that can be found within the dark genome [22]. They are poorly annotated for several reasons. Firstly, they arise sporadically via horizontal gene transfer, and consequently their distribution is unpredictable [7, 22]. Additionally, some uncharacterized EVE loci may be hard to recognize due to their being highly degraded or fragmented or because their exogenous virus counterparts are either unknown or extinct [92, 93]. Finally, there are numerous potential sources of confounding or artefactual results that can arise during EVE screening, including host genes that exhibit similarity to virus genes, and contamination of WGS assemblies with DNA derived from other sources, including exogenous viruses.

To illustrate how DIGS facilitates identification and characterization of features hidden within the dark genome, we used the DIGS tool to perform a broad-based investigation of EVE diversity in vertebrates. We first focussed on high-copy number EVEs—which in vertebrate genomes mainly comprise ERVs. We screened 874 vertebrate genomes for RT-encoding ERVs and identified 702,167 high confidence matches. This screen revealed marked differences in ERV RT copy number between vertebrate classes. An in-depth investigation of ERV diversity in vertebrates—for example, examining their composition in finer detail or incorporating insertions that lack RT sequences, was considered beyond the scope of this study. However, the RT dataset generated here provides a robust foundation for further ERV studies that are underpinned by phylogenetic analysis. For example, we have previously used RT data in combination with other in silico approaches for in-depth, phylogenetical characterization of ERVs within discrete mammalian subgroups (e.g., see [38]).

ERVs constitute an unusual type of EVE, in that they can remain replication-competent following integration and may increase their germline copy number through continued replication as viruses or TEs [94]. However, the germline copy number of any EVE can potentially increase through interactions with TEs—this has been described for ERVs [48, 95, 96], as well as for EVEs derived from DNAds viruses [59, 61, 62]. In addition, data obtained here and in our previous investigations show that EVEs derived from hepadnaviruses have been amplified in cormorants [35], while circovirus-derived sequences have been amplified in carnivore genomes [36], apparently in association with LINE1 activity [63]. Fusion between EVEs and vertebrate transposons has notably influenced vertebrate genome evolution—it has occurred on multiple independent occasions and involves a diverse range of vertebrate viruses. Interestingly, our investigations of LINE1-associated circovirus EVEs in carnivore genomes suggested that LINE1-like retroelements have also been incorporated into gammaherpesvirus genomes and possibly even into Chikungunya virus (Additional file 10: Fig. S8). These findings suggest that retroelement-mediated transposition can establish a complex network of horizontal gene transfer events linking virus and transposon genomes with those of their vertebrate hosts.

DIGS is well-suited to exploring the distribution and diversity of high copy number genome features such as ERVs and TEs but can also be used in “beach combing” searches of WGS data sets that aim to identify rare and unusual genome features. These kinds of screens typically require a rigorous filtering process to distinguish genuine from spurious matches, and as shown here, this is facilitated by database integration. DIGS enabled the efficient identification of EVEs derived from non-retroviral viruses (which are relatively rare and diverse) and provided a powerful framework for filtering spurious results (Additional file 3: Fig. S3).

Via DIGS, we established a broad overview of non-retroviral EVE diversity in vertebrate genomes (Table 1, Figs. 4 and 6), shedding new light on virus distribution and diversity in vertebrates. Notably, our findings extend the known host range of important virus families. For example, we identify a filovirus-derived EVE in a frog (order Anura), providing the first evidence for the existence of amphibian filoviruses. In addition, we provide the first evidence for the presence (at least historically) of hepadnaviruses in sharks and chuviruses in placental mammals (Fig. 4). In addition, we reveal novel virus diversity. For example, we identify novel lineages of parvoviruses and circoviruses in amphibians, as well as a novel circovirus lineage in turtles and a novel hepadnavirus lineage in frogs. We also identify novel paramyxovirus, chuvirus and bornavirus lineages in fish and amphibians.

Mammalian filoviruses include some of the most lethal viruses in the world [97], and while the natural reservoirs of some are known, they remain unclear for the highly pathogenic ebolavirus (EBOV) and its closest relatives (Fig. 5). EBOV is assumed to have a zoonotic origin, but it has rarely been possible to formally link outbreaks to a given animal reservoir, limiting understanding of its emergence. So far, efforts to identify the true reservoirs of ebolaviruses have tended to focus on bats [81]. However, the widespread presence of filovirus EVEs in rodents [63], including some groups that have not been examined as potential EBOV reservoirs, such as spiny mice, suggests that the potential of this group to serve as a reservoir should not be overlooked.

Previous studies have noted that filovirus EVE sequences in the genomes of cricetid rodents (family Cricetidae) robustly split the *Ebolavirus* and *Cuevavirus* genera from the *Marburgvirus* and *Dianlovirus* genera, demonstrating that these groups diverged > 20 million years ago (Mya) [98], rather than within the past 10,000 years as suggested by molecular clock-based analysis of contemporary filovirus genomes [99]. Here, we found that TAPV, an exogenous virus of snakes, robustly separates two clades of mammalian filoviruses in phylogenetic reconstructions. Since transmission of filoviruses between reptiles and mammals is likely quite rare, and both lineages contain ancient EVEs (Fig. 5, Additional file 9: Table S2), these findings support the long-term existence of two highly distinct filovirus lineages in mammals, which we labeled “mammal 1” and “mammal 2”. Notably, basal taxa within the “mammal 1” lineage—which also includes all known contemporary filoviruses of mammals—disclose associations with Southern Hemisphere continents (Australia, South America) that were largely isolated throughout extensive periods of the Cenozoic Era. These data suggest that filoviruses were present in ancestral mammals inhabiting Gondwanaland (an ancient supercontinent comprised of South America, Africa, India, and Australia) and diversified into at least two major lineages as mammalian populations became compartmentalized in distinct continental regions during the early to mid-Cenozoic. An interesting question is whether the “mammal 2” group represents filoviruses that evolved in Northern hemisphere-associated, boreoeutherian mammals (magnorder Boreoeutheria), while “mammal 1” represents filoviruses that initially evolved in Southern hemisphere-associated marsupials (infraclass Marsupialia) and xenarthrans (magnorder Xenarthra) before disseminating throughout the globe (possibly in association with volant mammals—i.e., bats).

While several previous studies have described EVE diversity in vertebrates [50, 53, 100], our investigation is significantly larger in scale and breadth. Furthermore, for non-retroviral viruses, we introduced a higher level of order to EVE data, making use of the DIGS framework to discriminate orthologous versus paralogous EVE loci and to identify intra-genomically amplified EVE lineages. This allowed us to establish a panoramic view of germline incorporation by non-retroviral viruses during vertebrate evolution (Fig. 7). Furthermore, discriminating orthologous and paralogous EVEs enabled us to infer the rates of germline infiltration by non-retroviral virus families with greater accuracy than in previous studies (Figs. 6 and 7). Notably, we did not find strong evidence for a reduced rate of germline infiltration in avian genomes, as suggested by a previous study [101]. Incorporation of DNArt viruses is higher in birds than in any other vertebrate class (Fig. 6), and while acquisition of EVEs derived from ssRNA-ve viruses does appear to be limited in this group, they closely resemble reptiles in this respect. Avian hosts also appear similar overall to reptiles with regard to ERV RT copy number (Fig. 3).

The absence, or near absence, of many virus groups from our catalog of vertebrate EVEs is noteworthy. For example, many distinct families of ssRNA + ve viruses infect vertebrates [65], but of these, only flaviviruses appear to have generated any EVEs (Fig. 4), and these only occur quite rarely compared to other virus groups (Table 3). Furthermore, EVEs derived from viruses with circular RNA genomes, or double-stranded RNA genomes, were not detected at all. EVEs derived from all other virus genome types do occur in the vertebrate germline, but their distribution is patchy and limited to a relatively small number of virus families (Figs. 4 and 7). For example, among ssRNA-ve viruses, only mononegaviruses were detected, with no evidence for germline integration of segmented ssRNA-ve viruses such as orthomyxoviruses and bunyaviruses. The limited presence of EVEs originating from specific vertebrate virus groups within vertebrate genomes implies that certain aspects of these groups’ biology in vertebrate hosts restrict their ability to be integrated into the germline. These aspects likely include cell tropism (whether germline cells are typically infected) and the site of cellular replication (with viruses that replicate in the nucleus being more likely to be incorporated) [21]. Additionally, vertebrate germline cells may present strong intrinsic barriers to the replication of certain virus groups.

The most ancient EVE identified in our study predates the divergence of birds and reptiles, nearly 300 Mya. More ancient EVEs will likely be difficult to identify due to sequence degradation. However, it is conceivable that progress in genome sequencing, EVE screening and virus discovery will enable the implementation of more sensitive screens that yield even older EVEs, potentially predating the emergence of vertebrates.

Besides identifying EVEs, our screen identified several sequences that appeared likely to derive from exogenous viruses (Additional file 5: Table S1). These overwhelmingly represented DNA virus families that contain at least some species that are capable of establishing chronic, latent infections and/or integrating into host cell chromosomes [102–104]. Potentially, the occurrence of contaminating DNA derived from specific exogenous virus groups in WGS data might serve as an indication of their tendency to establish chronic or latent infections. Our screen also uncovered virus-like sequences that seemed likely to derive from diet-related contamination of WGS data, either by viruses or EVEs (see Additional file 3: Fig. S3). It is worth noting that, in our data, these sequences stood out as potential contaminants because they derived from virus groups that infect plants, not animals (e.g., *Geminiviridae*, *Potyviridae*). However, similar contaminants might be more difficult to identify if they derived from animal viruses or EVEs, as may be expected to occur in diet-related contamination of WGS assemblies of carnivorous or insectivorous animal species.

The catalog of EVE loci generated here provides a foundation for further investigations in virology, genomics, and human health. From the virology perspective, EVEs provide information about the long-term evolutionary history viruses, which greatly influences how we understand their biology. As well as enabling future studies of vertebrate “paleoviruses”, the EVE catalog can inform efforts to identify and characterize new viruses (both by providing ecological and evolutionary insights [76] and by helping identify “false positive” hits arising from genomic DNA) [105].

From the genomics side, EVEs are of interest due to their important roles in physiology and genome evolution [106]. These include roles in antiviral immunity [11, 107, 108] as well as a diverse range of other physiological processes [18, 83, 84, 109–112]. Notably, we identified numerous non-retroviral EVEs encoding ORFs longer than 300 aa (Additional file 7: Fig. S6), indicating that their coding capacity has been conserved during vertebrate evolution. One of these—a chuvirus-derived L-protein identified in livebearers—adds to previous evidence that viral RdRp sequences have been co-opted by vertebrate genomes [71]. Mapping of EVE loci can also inform efforts to develop new medical treatments—in a recent study, EVE loci identified using DIGS were used to identify potential genomic safe harbors for human transgene therapy applications [33].

The EVE screen performed here has several important limitations. Firstly, it relied on published WGS data generated for extant species. Secondly, our results have likely been influenced by aspects of our screening configuration, such as the composition of the probe set with respect to viral taxa and polypeptide probe length [113, 114]. This might mean that we failed to detect some of the potentially recognizable EVE loci present in our TDb. For example, counts of RT-encoding ERV loci were found to be generally lower in ray-finned fish and jawless fish (Fig. 3), but previous studies have shown that RT loci related to other families of reverse-transcribing virus, such as metaviruses (family *Metaviridae*) [115] and “lokiretroviruses” [116] are relatively common in these hosts. These would likely have been missed in our search because they were not included in our RT RSL. Finally, previous studies have indicated that vertebrate genomes contain EVEs that lack any clear homology to extant viruses [117], and these would not be detected using a sequence similarity-based approach.

As vertebrate genome sequencing progresses, further opportunities to identify novel EVEs will arise, since: (i) any novel genome could in theory contain a lineage-specific EVE and (ii) ongoing characterization of exogenous virus diversity may allow for detection of previously undetectable EVEs, by providing new probe sequences. The DIGS project created here, which is openly available online, can be reused to accommodate newly sequenced vertebrate genomes (TDb expansion) and newly discovered vertebrate virus diversity (RSL/probe set expansion). In addition, similar projects can readily be created to screen for EVEs in other host groups.

The use of DIGS is not limited to investigations of EVEs. DIGS can be used to investigate any sufficiently conserved genome feature lurking within the dark genome, including both coding and non-coding elements (Table 1). Many of the most interesting genes have evolved relatively rapidly and are difficult to annotate reliably using automated approaches [118]. Furthermore, even relatively conserved genes may be incompletely annotated by automated pipelines. DIGS has previously been used to broadly survey the distribution of interferon stimulated genes in mammals [30] and for in-depth investigation of specific genes and gene families, such as OAS1 [27] and APOBEC3 [28]. While DIGS is best suited to investigations of genome features that comprise a single contiguous unit and contain relatively long, easily recognized regions, it can also be used to investigate genome features that are shorter or are comprised of several short sub-components, providing that a careful approach is used. For example, when investigating interferon lambda (IFNL) genes, which are expressed from multiple, short exons, we included conserved flanking features in our RSL and probe set [30] (Table 1). This enabled more confident matching of IFNL exons based on their positional relationships relative to conserved markers. We have also used DIGS in functional genomics studies to investigate the locations of short nucleotide motifs identified in binding assays (e.g., CHiP-seq) relative to other genomic features such as ERVs [25, 26].

The framework described here for implementing DIGS could be further developed and improved, for example, by including the option to use other sequence similarity search tools, such as Diamond [119] and ElasticBLAST [120], or RNA structure based search tools such as INFERNAL [121]. Integrating with functional genomics resources could provide further dimensionality to the kinds of investigations that may be performed using DIGS [122].

## Conclusions

We demonstrate how a relational database management system can be linked to a similarity search-based screening pipeline to investigate the dark genome in silico. Using this approach, we catalog and analyze EVEs throughout vertebrate genomes, providing a broad range of novel insights into the evolution of ancient viruses and their interactions with host species.

## Materials and methods

### Whole genome sequence and taxonomic data

Whole genome shotgun (WGS) sequence assemblies of 874 vertebrate species were obtained from the NCBI genomes resource [123]. Taxonomic data for the vertebrate species included in our screen and the viruses in our reference sequence library were obtained from the NCBI taxonomy database [124], using PERL scripts included with the DIGS tool package.

### Database-integrated screening for RT-encoding ERVs

An RT RSL was collated to represent diversity within the Retroviridae. We included representatives of previously identified ERV lineages and exogenous retrovirus species. A subset of these sequences was used as probes in similarity search-based screens [63]. For initial screening, we used a bitscore cutoff of 60. For comparisons of ERV RT copy number across species we filtered initial results using a more conservative bitscore cutoff of 90. Our previous, DIGS-based studies of ERVs have shown that spurious matches (i.e., to sequences other than retroviral RTs) do not arise when this cutoff is applied, although some genuine ERV RT hits may be excluded [38].

### Database-integrated screening for non-retroviral EVEs

We obtained an RSL representing the proteome of eukaryotic viruses from the NCBI virus genomes database [56]. We supplemented this with sequence likely to cross-match to virus probes during screening. These included the teratorn transposon found in fish, which contains multiple alloherpesvirus-derived genes [125]. We included the polypeptide sequences of these genes, obtained from the subtype 1 Teratorn reference (Accession #: LC199500) in our RSL. We also included representatives of the maverick/polinton lineage of transposons, derived from sequences defined in a previous study, since these elements are now recognized to derive from a group of midsize eukaryotic linear DNAds viruses referred to as “polinton-like viruses” or “adintoviruses” [59–61]. Probes constituted a subset of 685 sequences contained within our RSL and incorporated polypeptide sequences representing all major protein-coding genes of representative species of all recognized or provisional vertebrate virus families. We used a bit score cutoff of 60 as a threshold for counting non-retroviral EVE loci. This threshold was established through previous experience searching for non-retroviral EVEs using DIGS [31, 32, 35, 36]. Experience from previous studies had shown that nearly 100% of matches with bit scores ≥ 60 were either virus-derived or represented genuine similarity between virus genes and their cellular orthologs. By contrast, investigation of a subset of 100 hits with bit scores of b 40–59 showed that ~ 50% could not be confidently confirmed as having a viral origin (data not shown).

Artefactual hits to host DNA can occur in EVE screens since some virus genomes contain genes that have cellular homologs [126], and some virus genomes contain captured host DNA [127]. To distinguish host from virus-derived DNA in these cases, we exported such hits from the screening database and virtually translated them to obtain a polypeptide sequence. We then used the translated sequences as query input to online BLAST searches of GenBank’s non-redundant (nr) database. If searches revealed closer matching to host genes than to known viral genes, the input sequences were assumed to be host derived. Wherever this occurred, we incorporated representatives of the matching host sequences into the RSL, so that they would be recognized as host hits on reclassification. By updating hit classifications in this way, we could progressively filter out host-derived hits from our final screening output.

### Filtering sequences-derived from exogenous viruses

Sequences derived from exogenous viruses are occasionally incorporated into WGS assemblies. We used SQL queries to identify and exclude these sequences based on hit characteristics. Where hits derived from virus species or species groups that have been sequenced previously, they could be discriminated on the basis of sequence identity (i.e., 98–99% nucleotide-level identity known viruses. The “extract start” field could be used to identify sequences that lacked flanking genomic sequences, indicating a potential exogenous origin. We also examined the virtually translated sequences to look for evidence of long-term presence in the host germline (e.g., stop codons, frameshifting mutations).

### Filtering of cross-matching retrovirus-derived sequences

Hits that match more closely to virus genomes than to host DNA, and are clearly inserted into host DNA, are most likely *bona fide* EVE sequences. However, they may not necessarily be non-retroviral EVEs because some filoviruses and arenaviruses (family Arenaviridae) contain glycoprotein genes that are distantly related to those found in certain retroviruses [128, 129]. When such hits were investigated and found to correspond to ERVs (established through the presence of proviral genome features adjacent to the hit), we included the putative sequences of glycoproteins encoded by these ERVs into our RSL and reclassified hits, so that spurious matches could be recognized as ERV-derived.

### Genomic analysis

Previous studies of presence/absence patterns have shown that non-retroviral EVEs are present in many genomes due to orthology (ancient insertions) rather than paralogy (recent independent insertion) [32, 35, 36, 77]. To differentiate orthologs of previously described EVEs from newly identified paralogs, we expanded our RSL to include consensus/reference sequences representing unique EVE loci. This set of EVE loci was comprised of insertions identified in previous studies [32, 35, 36, 78, 130], as well as a set of clearly novel EVEs identified in the present screen. For high-copy number, amplified lineages within this set (see Additional file 9: Fig. S8), we only included a single reference sequence, rather than attempting to represent each individual ortholog, since it was clear that these elements derive from a single germline incorporation event. EVEs were considered novel if: (i) they derived from a virus group not previously reported in the host group in which they were identified or (ii) occurred in species only distantly related to species in which similar EVEs had been identified previously (e.g., an entirely distinct host class). Whenever novel EVEs were defined, results were reclassified using the updated RSL (see Fig. 2). Orthologs of previously identified EVEs could be inferred by using SQL queries to summarize screening results, as they disclosed high similarity to these EVE sequences and occurred in host species relatively closely related to the species in which the putatively orthologous EVEs had previously been identified. By contrast, novel paralogs either disclosed only limited similarity to previously identified EVE sequences or occurred in distantly related host species. This approach to discriminating between paralogs and orthologs has limitations but can guide further investigations that use more reliable approaches (e.g., via investigation of flanking sequences, or phylogeny) to infer orthology [35]. Se-Al (version 2.0a11) was used to inspect multiple sequence alignments of EVEs and genomic flanking sequences. Minimum age estimates were obtained for orthologous EVEs by using host species divergence time estimates collated in TimeTree [89]. We identified open reading frames and open coding regions within EVEs using PERL scripts available on request.

### Phylogenetic analysis

Phylogenies were reconstructed using the maximum likelihood approach implemented in RAxML (version 8.2.12) [131] and model parameters selected using IQ-TREE model selection function [132]. Support for phylogenies was assessed via 1000 non-parametric bootstrap replicates. A time-calibrated vertebrate phylogeny was obtained via TimeTree, an open database of species divergence time estimates [89]. To determine germline infiltration rate, we divided the total number of distinct EVE orthologs identified in each vertebrate class by the total amount of branch length sampled for that class (obtained from the time-calibrated phylogeny).

### Application of standardized nomenclature to EVE loci

We assigned all non-retroviral EVEs identified in our study unique identifiers (IDs), following a convention developed for ERVs [133]. Each was assigned a unique identifier (ID) constructed from three components. The first component is a classifier denoting the type of EVE. The second component comprises: (i) the name of the taxonomic group of viruses the element derived from and (ii) a numeric ID that uniquely identifies a specific integration locus, or for multicopy lineages, a unique founding event. The final component denotes the taxonomic distribution of the element. This approach has been applied in several previous studies of vertebrate EVEs [31, 32, 35, 78] and we maintained consistency with these studies with respect to the numeric ID. Where our study revealed new information about the taxonomic relationship of an EVE to contemporary viruses, or its distribution across taxa, the ID was updated accordingly.

### Supplementary Information

**Additional file 1: Figure S1.** An annotated example of a DIGS tool control file.

**Additional file 2: Figure S2.** The DIGS tool framework for in silico genome screening.

**Additional file 3: Figure S3.** Examples of SQL-based querying of DIGS results.

**Additional file 4: Figure S4.** Validation of the DIGS tool.

**Additional file 5: Table S1.** Putatively exogenous viruses identified in WGS data.

**Additional file 6: Figure S5.** Genomic analysis of a superficially caulimovirus-like EVE.

**Additional file 7: Figure S6.** Summary of vertebrate EVE coding potential.

**Additional file 8: Figure S7.** Evolutionary relationships of vertebrate EVEs and viruses.

**Additional file 9: Figure S8.** Amplified lineages of endogenous viral elements.

**Additional file 10: Table S2.** Minimum ages of EVEs.

**Additional file 11: Figure S9.** Germline incorporation through time shown separately for each virus family.

**Additional file 12.** Review history.

## Data Availability

Source code for the DIGS tool is freely available under the GNU AGPL-3.0 license: GitHub: https://github.com/giffordlabcvr/DIGS-tool [134] Zenodo: 10.5281/zenodo.10948938 [135] All data generated in this study are openly available via GitHub: https://github.com/giffordlabcvr/DIGS-for-EVEs [136]

## References

[1] Margulies EH, Birney E (2008). Approaches to comparative sequence analysis: towards a functional view of vertebrate genomes. Nat Rev Genet.

[2] Cheng JF, Priest JR, Pennacchio LA (2007). Comparative genomics: a tool to functionally annotate human DNA. Methods Mol Biol.

[3] Nobrega MA, Pennacchio LA (2004). Comparative genomic analysis as a tool for biological discovery. J Physiol.

[4] Guan D, Lazar MA (2019). Shining light on dark matter in the genome. Proc Natl Acad Sci U S A.

[5] Wright BW (2022). The dark proteome: translation from noncanonical open reading frames. Trends Cell Biol.

[6] Eisenstein M (2021). Drug hunters uncloak the non-coding ‘hidden’ genome. Nat Biotechnol.

[7] Katzourakis A, Gifford RJ (2010). Endogenous viral elements in animal genomes. PLoS Genet.

[8] Chiba S (2011). Widespread endogenization of genome sequences of non-retroviral RNA viruses into plant genomes. PLoS Pathog.

[9] Diop SI (2018). Tracheophyte genomes keep track of the deep evolution of the Caulimoviridae. Sci Rep.

[10] Soucy SM, Huang J, Gogarten JP (2015). Horizontal gene transfer: building the web of life. Nat Rev Genet.

[11] Parrish NF, Tomonaga K (2016). Endogenized viral sequences in mammals. Curr Opin Microbiol.

[12] de Tomás C, Vicient CM (2022). Genome-wide identification of reverse transcriptase domains of recently inserted endogenous plant pararetrovirus (Caulimoviridae). Front Plant Sci.

[13] Gong Z, Zhang Y, Han GZ (2020). Molecular fossils reveal ancient associations of dsDNA viruses with several phyla of fungi. Virus Evol.

[14] Bellas C (2023). Large-scale invasion of unicellular eukaryotic genomes by integrating DNA viruses. Proc Natl Acad Sci U S A.

[15] Dewannieux M, Heidmann T (2013). Endogenous retroviruses: acquisition, amplification and taming of genome invaders. Curr Opin Virol.

[16] Geis FK, Goff SP (2020). Silencing and transcriptional regulation of endogenous retroviruses: an overview. Viruses.

[17] SrinivasacharBadarinarayan S, Sauter D (2021). Switching sides: how endogenous retroviruses protect us from viral infections. J Virol.

[18] Fujino K (2021). A human endogenous bornavirus-like nucleoprotein encodes a mitochondrial protein associated with cell viability. J Virol.

[19] Ophinni Y (2019). piRNA-guided CRISPR-like immunity in eukaryotes. Trends Immunol.

[20] Patel MR, Emerman M, Malik HS (2011). Paleovirology - ghosts and gifts of viruses past. Curr Opin Virol.

[21] Holmes EC (2011). The evolution of endogenous viral elements. Cell Host Microbe.

[22] Feschotte C, Gilbert C (2012). Endogenous viruses: insights into viral evolution and impact on host biology. Nat Rev Genet.

[23] Altschul SF (1997). Gapped BLAST and PSI-BLAST: a new generation of protein database search programs. Nuc Acids Res.

[24] Camacho C (2009). BLAST+: architecture and applications. BMC Bioinformatics.

[25] Fernandes LP (2022). A satellite DNA array barcodes chromosome 7 and regulates totipotency via ZFP819. Sci Adv.

[26] Enriquez-Gasca R (2023). Co-option of endogenous retroviruses through genetic escape from TRIM28 repression. Cell Rep.

[27] Wickenhagen A (2021). A prenylated dsRNA sensor protects against severe COVID-19. Science.

[28] Ito J, Gifford RJ, Sato K (2020). Retroviruses drive the rapid evolution of mammalian APOBEC3 genes. Proc Natl Acad Sci U S A.

[29] Shaw AE (2017). Fundamental properties of the mammalian innate immune system revealed by multispecies comparison of type I interferon responses. PLoS Biol.

[30] Bamford CGG, et al. Partial gene conversion shapes the emergence of functional novelty in the placental mammal interferon lambda system. In: Infectious diseases through an evolutionary lens. London: British Medical Association House; 2023.

[31] Bamford CGG (2022). Comparative analysis of genome-encoded viral sequences reveals the evolutionary history of flavivirids (family Flaviviridae). Virus Evol.

[32] Campbell MA, Loncar S, Kotin RM, Gifford RJ. Comparative analysis reveals the long-term coevolutionary history of parvoviruses and vertebrates. PLoS Biol. 2022;20(11):e3001867. 10.1371/journal.pbio.3001867. 10.1371/journal.pbio.3001867 PMC9707805 36445931

[33] Quezada-Ramírez MA, et al. Identification of genome safe harbor loci for human gene therapy based on evolutionary biology and comparative genomics. bioRxiv. 2023:2023.09.08.556857.

[34] Callaway HM (2019). Examination and reconstruction of three ancient endogenous parvovirus capsid protein gene remnants found in rodent genomes. J Virol.

[35] Lytras S, Arriagada G, Gifford RJ (2021). Ancient evolution of hepadnaviral paleoviruses and their impact on host genomes. Virus Evol.

[36] Dennis TPW (2019). The evolution, distribution and diversity of endogenous circoviral elements in vertebrate genomes. Virus Res.

[37] Kambol R, Gatseva A, Gifford RJ (2022). An endogenous lentivirus in the germline of a rodent. Retrovirology.

[38] Zhu H, Gifford RJ, Murcia PR (2018). Distribution, diversity, and evolution of endogenous retroviruses in perissodactyl genomes. J Virol.

[39] Blanco-Melo D, Gifford RJ, Bieniasz PD (2017). Co-option of an endogenous retrovirus envelope for host defense in hominid ancestors. Elife.

[40] Blanco-Melo D, Gifford RJ, Bieniasz PD (2018). Reconstruction of a replication-competent ancestral murine endogenous retrovirus-L. Retrovirology.

[41] Pearson WR, Mackey AJ (2017). Using SQL databases for sequence similarity searching and analysis. Curr Protoc Bioinformatics.

[42] Belyi VA, Levine AJ, Skalka AM (2010). Sequences from ancestral single-stranded DNA viruses in vertebrate genomes: the parvoviridae and circoviridae are more than 40 to 50 million years old. J Virol.

[43] Heusinger E (2015). Early vertebrate evolution of the host restriction factor tetherin. J Virol.

[44] Blanco-Melo D, Venkatesh S, Bieniasz PD (2016). Origins and evolution of tetherin, an orphan antiviral gene. Cell Host Microbe.

[45] Waterhouse RM (2013). OrthoDB: a hierarchical catalog of animal, fungal and bacterial orthologs. Nucleic Acids Res.

[46] Cunningham F (2015). Ensembl 2015. Nucleic Acids Res.

[47] Gifford RJ. Database-integrated genome screening (DIGS) tool. 2022. Available from: https://giffordlabcvr.github.io/DIGS-tool/.

[48] Belshaw R (2005). High copy number in human endogenous retrovirus families is associated with copying mechanisms in addition to reinfection. Mol Biol Evol.

[49] Johnson WE (2019). Origins and evolutionary consequences of ancient endogenous retroviruses. Nat Rev Microbiol.

[50] Hayward A, Grabherr M, Jern P (2013). Broad-scale phylogenomics provides insights into retrovirus-host evolution. Proc Natl Acad Sci U S A.

[51] Xiong Y, Eickbush TH (1990). Origin and evolution of retroelements based upon their reverse transcriptase sequences. EMBO J.

[52] Tristem M (2000). Identification and characterization of novel human endogenous retrovirus families by phylogenetic screening of the human genome mapping project database. J Virol.

[53] Hayward A, Cornwallis CK, Jern P (2015). Pan-vertebrate comparative genomics unmasks retrovirus macroevolution. Proc Natl Acad Sci U S A.

[54] Han GZ (2015). Extensive retroviral diversity in shark. Retrovirology.

[55] Xu X (2018). Endogenous retroviruses of non-avian/mammalian vertebrates illuminate diversity and deep history of retroviruses. PLoS Pathog.

[56] Brister JR (2015). NCBI viral genomes resource. Nucleic Acids Res.

[57] Sharma V (2020). Large-scale survey reveals pervasiveness and potential function of endogenous geminiviral sequences in plants. Virus Evol.

[58] Tanne E, Sela I (2005). Occurrence of a DNA sequence of a non-retro RNA virus in a host plant genome and its expression: evidence for recombination between viral and host RNAs. Virology.

[59] Koonin EV, Krupovic M, Yutin N (2015). Evolution of double-stranded DNA viruses of eukaryotes: from bacteriophages to transposons to giant viruses. Ann N Y Acad Sci.

[60] Barreat JGN, Katzourakis A (2021). Phylogenomics of the Maverick virus-like mobile genetic elements of vertebrates. Mol Biol Evol.

[61] Starrett GJ (2021). Adintoviruses: a proposed animal-tropic family of midsize eukaryotic linear dsDNA (MELD) viruses. Virus Evol.

[62] Inoue Y, Takeda H (2023). Teratorn and its relatives - a cross-point of distinct mobile elements, transposons and viruses. Front Vet Sci.

[63] Gifford RJ. DIGS-for-EVEs. 2023. Available from: https://github.com/giffordlabcvr/DIGS-for-EVEs.

[64] Harvey E (2023). Divergent hepaciviruses, delta-like viruses and a chu-like virus in Australian marsupial carnivores (dasyurids). Virus Evol.

[65] Harvey E, Holmes EC (2022). Diversity and evolution of the animal virome. Nat Rev Microbiol.

[66] Ariel E (2011). Viruses in reptiles. Vet Res.

[67] Waller SJ (2022). Cloacal virome of an ancient host lineage - the tuatara (Sphenodon punctatus) - reveals abundant and diverse diet-related viruses. Virology.

[68] Soto E (2020). First isolation of a novel aquatic flavivirus from Chinook Salmon (Oncorhynchus tshawytscha) and its in vivo replication in a piscine animal model. J Virol.

[69] Koda SA (2021). Complete genome sequences of infectious spleen and kidney necrosis virus isolated from farmed albino rainbow sharks Epalzeorhynchos frenatum in the United States. Virus Genes.

[70] Harding EF (2022). Revealing the uncharacterised diversity of amphibian and reptile viruses. ISME Commun.

[71] Horie M (2016). An RNA-dependent RNA polymerase gene in bat genomes derived from an ancient negative-strand RNA virus. Sci Rep.

[72] Ho ALFC, Pruett CL, Lin J (2016). Phylogeny and biogeography of Poecilia (Cyprinodontiformes: Poeciliinae) across Central and South America based on mitochondrial and nuclear DNA markers. Mol Phylogenet Evol.

[73] Aswad A, Katzourakis A (2014). The first endogenous herpesvirus, identified in the tarsier genome, and novel sequences from primate rhadinoviruses and lymphocryptoviruses. PLoS Genet.

[74] Aswad A (2021). Evolutionary history of endogenous human herpesvirus 6 reflects human migration out of Africa. Mol Biol Evol.

[75] Liu X (2020). Endogenization and excision of human herpesvirus 6 in human genomes. PLoS Genet.

[76] Dennis TPW (2018). Insights into circovirus host range from the genomic fossil record. J Virol.

[77] Suh A (2014). Early mesozoic coexistence of amniotes and hepadnaviridae. PLoS Genet.

[78] Kawasaki J (2021). 100-My history of bornavirus infections hidden in vertebrate genomes. Proc Natl Acad Sci U S A.

[79] Horie M (2010). Endogenous non-retroviral RNA virus elements in mammalian genomes. Nature.

[80] Hyndman TH (2012). Isolation and molecular identification of Sunshine virus, a novel paramyxovirus found in Australian snakes. Infect Genet Evol.

[81] Mari Saez A (2015). Investigating the zoonotic origin of the West African Ebola epidemic. EMBO Mol Med.

[82] Leroy EM (2004). Multiple Ebola virus transmission events and rapid decline of central African wildlife. Science.

[83] Edwards MR (2018). Conservation of structure and immune antagonist functions of filoviral VP35 homologs present in microbat genomes. Cell Rep.

[84] Kondoh T (2017). Putative endogenous filovirus VP35-like protein potentially functions as an IFN antagonist but not a polymerase cofactor. PLoS One.

[85] Gorbalenya AE, Lauber C. Phylogeny of viruses. In: Reference module in biomedical sciences. 2017.

[86] Li Y (2022). Endogenous viral elements in shrew genomes provide insights into pestivirus ancient history. Mol Biol Evol.

[87] Qin XC (2014). A tick-borne segmented RNA virus contains genome segments derived from unsegmented viral ancestors. Proc Natl Acad Sci U S A.

[88] Krupovic M, Koonin EV (2015). Polintons: a hotbed of eukaryotic virus, transposon and plasmid evolution. Nat Rev Microbiol.

[89] Kumar S (2022). TimeTree 5: an expanded resource for species divergence times. Mol Biol Evol.

[90] Birney E (2007). Identification and analysis of functional elements in 1% of the human genome by the ENCODE pilot project. Nature.

[91] Schattner P (2007). Automated querying of genome databases. PLoS Comput Biol.

[92] Obbard DJ (2018). Expansion of the metazoan virosphere: progress, pitfalls, and prospects. Curr Opin Virol.

[93] Zhang YZ, Shi M, Holmes EC (2018). Using metagenomics to characterize an expanding virosphere. Cell.

[94] Koonin EV, Dolja VV (2014). Virus world as an evolutionary network of viruses and capsidless selfish elements. Microbiol Mol Biol Rev.

[95] Reus K (2001). HERV-K(OLD): ancestor sequences of the human endogenous retrovirus family HERV-K(HML-2). J Virol.

[96] Pavlícek A (2002). Processed pseudogenes of human endogenous retroviruses generated by LINEs: their integration, stability, and distribution. Genome Res.

[97] Mahanty S, Bray M (2004). Pathogenesis of filoviral haemorrhagic fevers. Lancet Infect Dis.

[98] Taylor DJ (2014). Evidence that ebolaviruses and cuevaviruses have been diverging from marburgviruses since the Miocene. PeerJ.

[99] Carroll SA (2013). Molecular evolution of viruses of the family Filoviridae based on 97 whole-genome sequences. J Virol.

[100] Kryukov K (2019). Systematic survey of non-retroviral virus-like elements in eukaryotic genomes. Virus Res.

[101] Cui J (2014). Low frequency of paleoviral infiltration across the avian phylogeny. Genome Biol.

[102] Osterrieder N, Wallaschek N, Kaufer BB (2014). Herpesvirus genome integration into telomeric repeats of host cell chromosomes. Annu Rev Virol.

[103] McBride AA, Warburton A (2017). The role of integration in oncogenic progression of HPV-associated cancers. PLoS Pathog.

[104] Janovitz T (2017). Parvovirus B19 integration into human CD36+ erythroid progenitor cells. Virology.

[105] Brait N (2023). A tale of caution: how endogenous viral elements affect virus discovery in transcriptomic data. Virus Evol.

[106] Frank JA, Feschotte C (2017). Co-option of endogenous viral sequences for host cell function. Curr Opin Virol.

[107] Aswad A, Katzourakis A (2012). Paleovirology and virally derived immunity. Trends Ecol Evol.

[108] Bravo A (2023). Antiviral activity of an endogenous parvoviral element. Viruses.

[109] Lavialle C (2013). Paleovirology of ‘syncytins’, retroviral env genes exapted for a role in placentation. Philos Trans R Soc Lond B Biol Sci.

[110] Valencia-Herrera I (2019). Molecular properties and evolutionary origins of a parvovirus-derived myosin fusion gene in guinea pigs. J Virol.

[111] Pastuzyn ED (2018). The neuronal gene arc encodes a repurposed retrotransposon gag protein that mediates intercellular RNA transfer. Cell.

[112] Koonin EV, Krupovic M (2018). The depths of virus exaptation. Curr Opin Virol.

[113] Hu G, Kurgan L (2019). Sequence similarity searching. Curr Protoc Protein Sci.

[114] Pearson WR (2013). An introduction to sequence similarity (“homology”) searching. Curr Protoc Bioinformatics.

[115] Miller K (1999). Identification of multiple Gypsy LTR-retrotransposon lineages in vertebrate genomes. J Mol Evol.

[116] Wang J, Han GZ (2021). A sister lineage of sampled retroviruses corroborates the complex evolution of retroviruses. Mol Biol Evol.

[117] Kojima S (2021). Virus-like insertions with sequence signatures similar to those of endogenous nonretroviral RNA viruses in the human genome. Proc Natl Acad Sci U S A.

[118] Bruno M, Mahgoub M, Macfarlan TS (2019). The arms race between KRAB-zinc finger proteins and endogenous retroelements and its impact on mammals. Annu Rev Genet.

[119] Buchfink B, Reuter K, Drost HG (2021). Sensitive protein alignments at tree-of-life scale using DIAMOND. Nat Methods.

[120] Camacho C (2023). ElasticBLAST: accelerating sequence search via cloud computing. BMC Bioinformatics.

[121] Nawrocki EP, Kolbe DL, Eddy SR (2009). Infernal 1.0: inference of RNA alignments. Bioinformatics.

[122] Grabowski P, Rappsilber J (2019). A primer on data analytics in functional genomics: how to move from data to insight?. Trends Biochem Sci.

[123] Kitts PA (2016). Assembly: a resource for assembled genomes at NCBI. Nucleic Acids Res.

[124] Schoch CL (2020). NCBI Taxonomy: a comprehensive update on curation, resources and tools. Database (Oxford).

[125] Inoue Y (2018). Fusion of piggyBac-like transposons and herpesviruses occurs frequently in teleosts. Zoological Lett.

[126] Koonin EV (2009). On the origin of cells and viruses: primordial virus world scenario. Ann N Y Acad Sci.

[127] Becher P, Tautz N (2011). RNA recombination in pestiviruses: cellular RNA sequences in viral genomes highlight the role of host factors for viral persistence and lethal disease. RNA Biol.

[128] Benit L, Dessen P, Heidmann T (2001). Identification, phylogeny, and evolution of retroviral elements based on their envelope genes. J Virol.

[129] Gallaher WR, DiSimone C, Buchmeier MJ (2001). The viral transmembrane superfamily: possible divergence of Arenavirus and Filovirus glycoproteins from a common RNA virus ancestor. BMC Microbiol.

[130] Hildebrandt E (2020). Evolution of dependoparvoviruses across geological timescales – implications for design of AAV-based gene therapy vectors. Virus Evol.

[131] Stamatakis A (2006). RAxML-VI-HPC: maximum likelihood-based phylogenetic analyses with thousands of taxa and mixed models. Bioinformatics.

[132] Minh BQ (2020). IQ-TREE 2: new models and efficient methods for phylogenetic inference in the genomic era. Mol Biol Evol.

[133] Gifford RJ (2018). Nomenclature for endogenous retrovirus (ERV) loci. Retrovirology.

[134] Blanco-Melo D, et al. DIGS-tool: database-integrated genome screening. Github; 2023. https://github.com/giffordlabcvr/DIGS-tool.

[135] Blanco-Melo D, et al. DIGS-tool version 1.0.4. Zenodo; 2024. https://zenodo.org/records/10948938.

[136] Blanco-Melo D, et al. DIGS datasets. Github; 2023. https://github.com/giffordlabcvr/DIGS-for-EVEs.

