# Supplementary material for: A novel approach to exploring the dark genome and its application to mapping of the vertebrate virus fossil record
Source: Genome Biol. 2024 May 13;25:120. doi: 10.1186/s13059-024-03258-y (PMC11089739; doi:10.1186/s13059-024-03258-y)
Supplement: Supplementary file 1 — Additional file 1: Figure S1. An annotated example of a DIGS tool control file. [file 13059_2024_3258_MOESM1_ESM.pdf]

```
# DIGS screening database connection parameters

Begin SCREENDB;
            db_name=eve_1_parvoviridae;
            mysql_server=localhost;
ENDBLOCK;
```

Screening DB connection details

```
# Paths and parameters for in silico screening using DIGS

BEGIN SCREENSETS;
            query_aa_fasta=/parvo-probes.faa;
            reference_aa_fasta=/parvo-refs.faa;
               output_path=./tmp/;
            bitscore_min_tblastn=60;
            seq_length_minimum=40;
            defragment_range=10;
            consolidate_range=3000;
            blast_threads=8;
ENDBLOCK;
```

— Path to the query sequences in FASTA format

— Path to reference sequence library (RSL)

Params

```
# List of target genomes to screen

BEGIN TARGETS;
            Agnatha/
            Actinopterygii/
            Sarcopterygii/
            Chondrichthyes/
            Amphibia/
            Squamata/
            Crocodilia/
            Aves/
            Mammalia/
ENDBLOCK;
```

Target database

Figure S1. An annotated example of a DIGS tool control file.

The DIGS tool control file defines parameters for screening and the paths to reference sequence library (RSL), probe, and target database (TDb) files. Control files are structured as 'NEXUS' style blocks {Maddison, 1997 #86} delineated by BEGIN and ENDBLOCK tokens.

References:

Maddison, D.R., D.L. Swofford, and W.P. Maddison, *NEXUS: an extensible file format for systematic information.* Syst Biol, 1997. **46**(4): p. 590-621.
