# Supplementary material for: A novel approach to exploring the dark genome and its application to mapping of the vertebrate virus fossil record
Source: Genome Biol. 2024 May 13;25:120. doi: 10.1186/s13059-024-03258-y (PMC11089739; doi:10.1186/s13059-024-03258-y)
Supplement: Supplementary file 2 — Additional file 2: Figure S2. The DIGS tool framework for in silico genome screening. [file 13059_2024_3258_MOESM2_ESM.pdf]

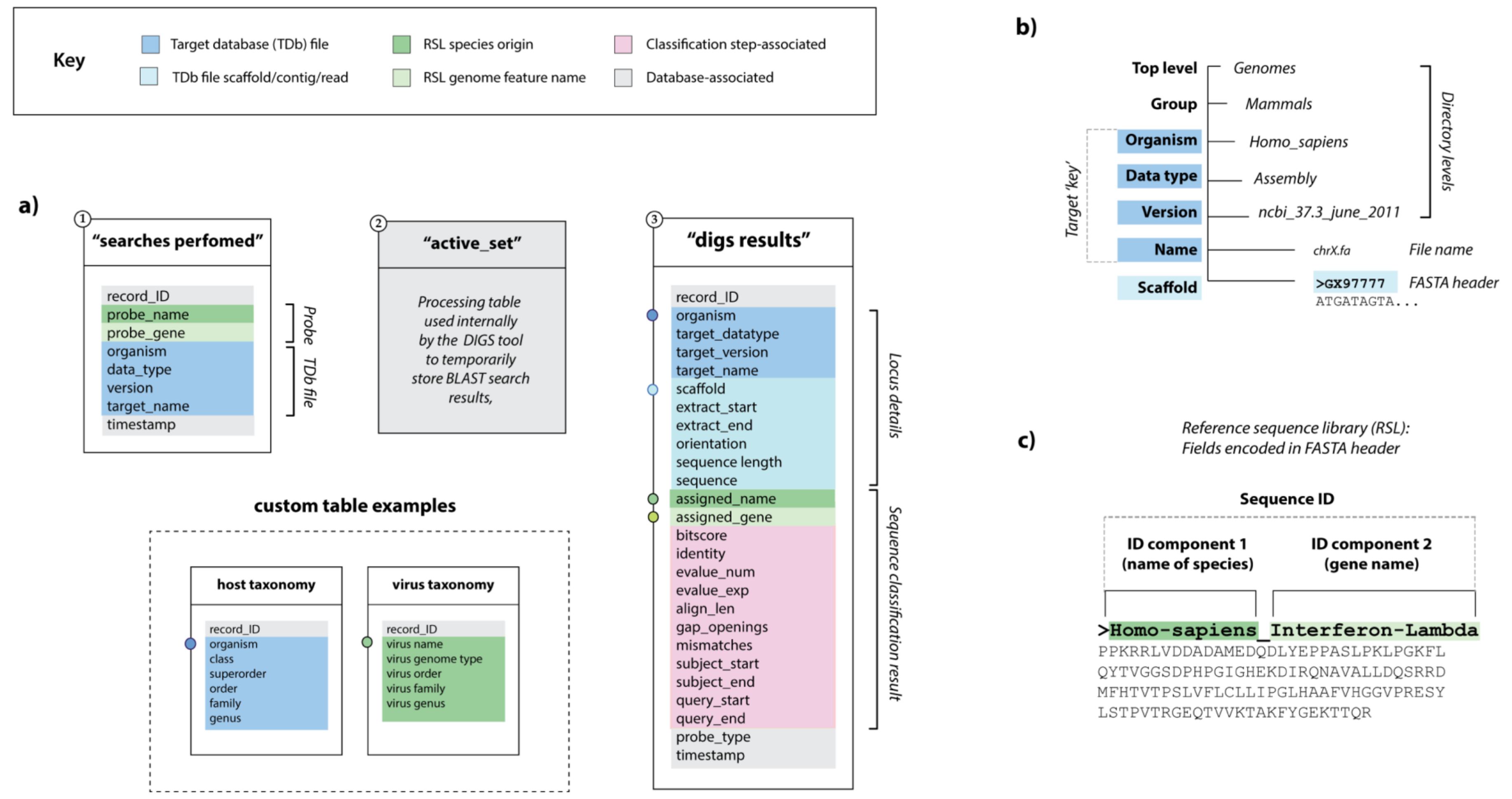

Figure S2. The DIGS tool framework for *in silico* genome screening.

Panels show schematic representations of key components of the *in silico* genome screening framework that is implemented in the database-integrated (DIGS) tool. Colours indicate relationships between components.

***(a) Screening database schema*.**

The database schema consists of three essential tables: (1) a status table ('searches performed'), (2) a data processing table ('active set'), and (3) a results table ('digs results').

These tables serve distinct purposes within the screening process:
Status Table: This table is used to monitor progress by keeping a record of the searches performed. It tracks progress in screening, by recording BLAST-based queries (i.e. which probes have so far been used to screen which TDb files).

Data Processing Table: The data processing table is employed by the DIGS screening algorithm. It temporarily stores and analyses the outcomes of individual BLAST searches and is cleared after each screening iteration.

Results Table: The results table holds a unique collection of all the hits obtained during the screening. It includes fields that define: (i) the location and orientation of a contiguous region of sequence within a specific genomic scaffold within a specific TDb file; (ii) the closest RSL match to each hit, as determined by BLAST comparison (see **Fig. 2** in main text), and associated values.

Additional flexibility is provided by the ability to expand DIGS screening databases with extra tables linked to the core schema using designated fields, as indicated in the figure by coloured circles. The inset box offers two examples of custom tables utilized in this study:

Virus Taxonomy Table: This table is linked via the 'assigned name' field.
Host Taxonomy Table: This table is linked via the 'organism' field.

(b) Target database directory hierarchy. The DIGS tool requires that TDb files are arranged in a directory hierarchy as shown. Directory levels correspond to screening database fields as shown in panel (a). Concatenating the values of four fields shown in darker blue (i.e. ‘organism’, ‘data type’, and ‘assembly version’, and ‘target name’) forms a unique identifier for target files. These fields define, respectively, the species of genome data origin, a user-defined data ‘type’ (e.g., ‘sanger genome’, ‘ngs-transcriptome’), assembly version name, and a specific genome assembly file. Extending this concatenation to include the ‘scaffold’, ‘extract start’ and ‘extract end’ fields defines a locus within the TDb.

(c) Reference sequence library. The reference sequence library (RSL) is a representative set of reference sequences for the genome feature(s) under investigation. FASTA headers encode two levels of classification; (i) the name of the species from which the sequence derives; (ii) the name of the genome feature represented by the sequence.
