# Supplementary material for: A novel approach to exploring the dark genome and its application to mapping of the vertebrate virus fossil record
Source: Genome Biol. 2024 May 13;25:120. doi: 10.1186/s13059-024-03258-y (PMC11089739; doi:10.1186/s13059-024-03258-y)
Supplement: Supplementary file 3 — Additional file 3: Figure S3. Examples of SQL-based querying of DIGS results. [file 13059_2024_3258_MOESM3_ESM.pdf]

a)

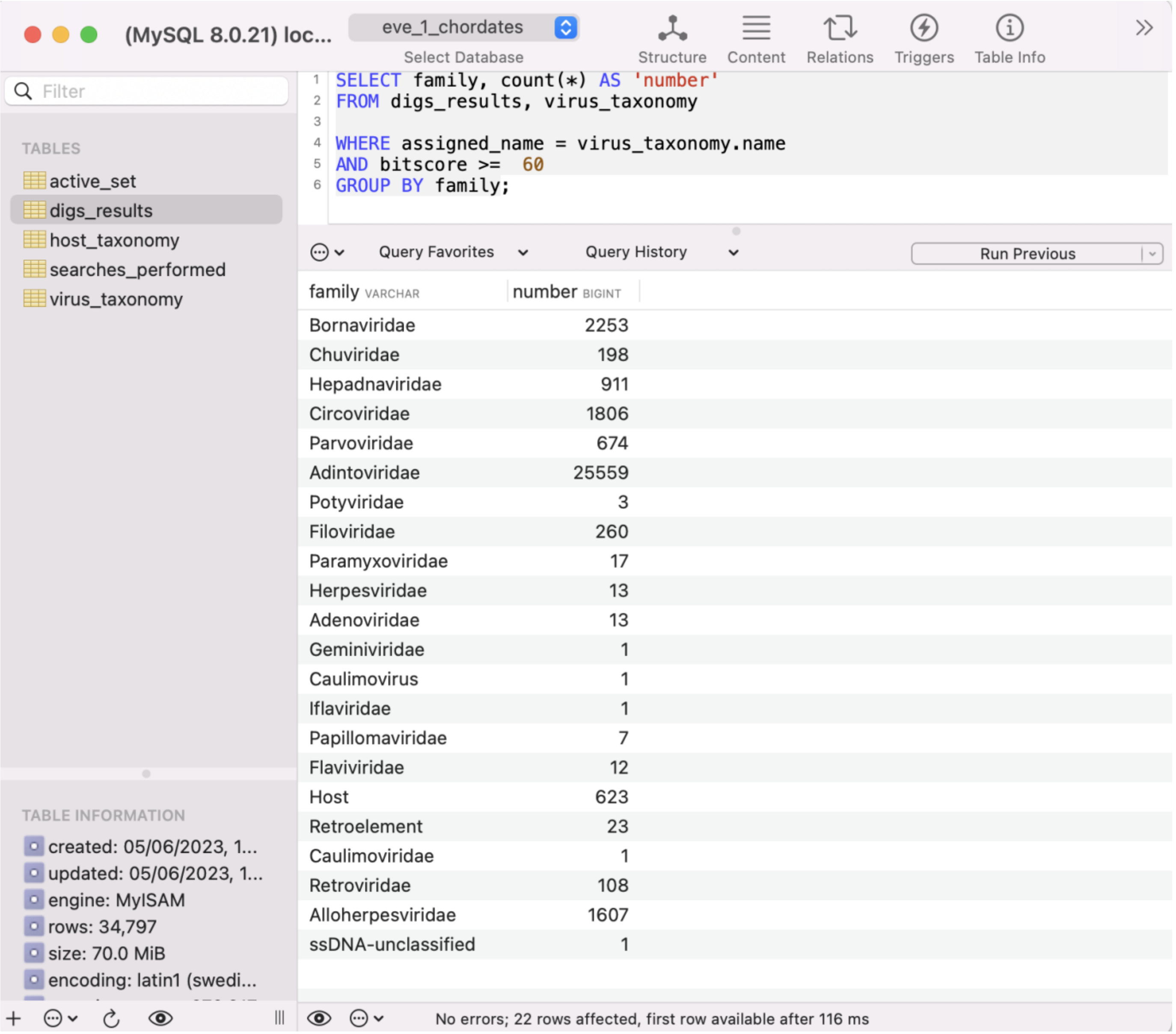


```
SELECT family, count(*) AS 'number'
FROM digs_results, virus_taxonomy

WHERE assigned_name = virus_taxonomy.name
AND bitscore >=  60
GROUP BY family;
```

family VARCHAR	number BIGINT
Bornaviridae	2253
Chuviridae	198
Hepadnaviridae	911
Circoviridae	1806
Parvoviridae	674
Adintoviridae	25559
Potyviridae	3
Filoviridae	260
Paramyxoviridae	17
Herpesviridae	13
Adenoviridae	13
Geminiviridae	1
Caulimovirus	1
Iflaviridae	1
Papillomaviridae	7
Flaviviridae	12
Host	623
Retroelement	23
Caulimoviridae	1
Retroviridae	108
Alloherpesviridae	1607
ssDNA-unclassified	1

b)

(MySQL 8.0.21) localhost/eve_1_chordates/digs_results

eve_1_chordates — Select Database

Structure | Content | Relations | Triggers | Table Info | Query | Table History | Users | Console

Filter

TABLES
- active_set
- digs_results
- host_taxonomy
- searches_performed
- virus_taxonomy

```sql
SELECT host_taxonomy.species, host_taxonomy.tax_class,  virus_taxonomy.family, assigned_name, assigned_gene, bitscore, identity
FROM  host_taxonomy,virus_taxonomy,  digs_results

WHERE virus_taxonomy.family = 'Paramyxoviridae'
AND bitscore >= 60
AND assigned_name = virus_taxonomy.name
AND organism = host_taxonomy.species

ORDER BY host_taxonomy.tax_class, host_taxonomy.superorder, host_taxonomy.tax_order, host_taxonomy.family, host_taxonomy.genus
```

Query Favorites | Query History | Run Current

species VARCHAR	tax_class VARCHAR	family VARCHAR	assigned_name VARCHAR	assigned_gene VARCHAR	bitscore	identity FLOAT
Nothobranchius_furzeri	Actinopteri	Paramyxoviridae	Tailam-virus	RNA-polymerase	260	39.211
Nothobranchius_furzeri	Actinopteri	Paramyxoviridae	Tailam-virus	RNA-polymerase	256	39.892
Nothobranchius_furzeri	Actinopteri	Paramyxoviridae	Avian-paramyxovirus-5	nucleoprotein	76.3	31.25
Astyanax_mexicanus	Actinopteri	Paramyxoviridae	Avian-paramyxovirus-5	nucleoprotein	74.7	25.309
Astyanax_mexicanus	Actinopteri	Paramyxoviridae	Avian-paramyxovirus-5	nucleoprotein	80.1	24.419
Astyanax_mexicanus	Actinopteri	Paramyxoviridae	Human-parainfluenza-virus-1	L-protein	312	41.162
Astyanax_mexicanus	Actinopteri	Paramyxoviridae	Avian-paramyxovirus-5	nucleoprotein	88.6	23.504
Astyanax_mexicanus	Actinopteri	Paramyxoviridae	Mojiang-virus	nucleocapsid	70.1	25
Astyanax_mexicanus	Actinopteri	Paramyxoviridae	Avian-paramyxovirus-5	nucleoprotein	78.2	26.22
Beryx_splendens	Actinopteri	Paramyxoviridae	Bovine-parainfluenza-virus-3	large-polymerase-subun...	122	29.084
Rondeletia_loricata	Actinopteri	Paramyxoviridae	Bovine-parainfluenza-virus-3	large-polymerase-subun...	120	44.828
Rondeletia_loricata	Actinopteri	Paramyxoviridae	Porcine-parainfluenza-virus-1	RNA-polymerase	82.8	40.385
Coryphaenoides_rupestris	Actinopteri	Paramyxoviridae	Mojiang-virus	polymerase	138	44.366
Scophthalmus_maximus	Actinopteri	Paramyxoviridae	Nipah-virus	polymerase	81.3	34.591
Limnodynastes_dumerilii	Amphibia	Paramyxoviridae	Sunshine_virus	RDRP	207	60.989
Limnodynastes_dumerilii	Amphibia	Paramyxoviridae	Sunshine_virus	RDRP	273	49.813
Scyliorhinus_torazame	Chondrichthyes	Paramyxoviridae	Sendai-virus	hemagglutinin-neuramin...	97.4	34.266

c)

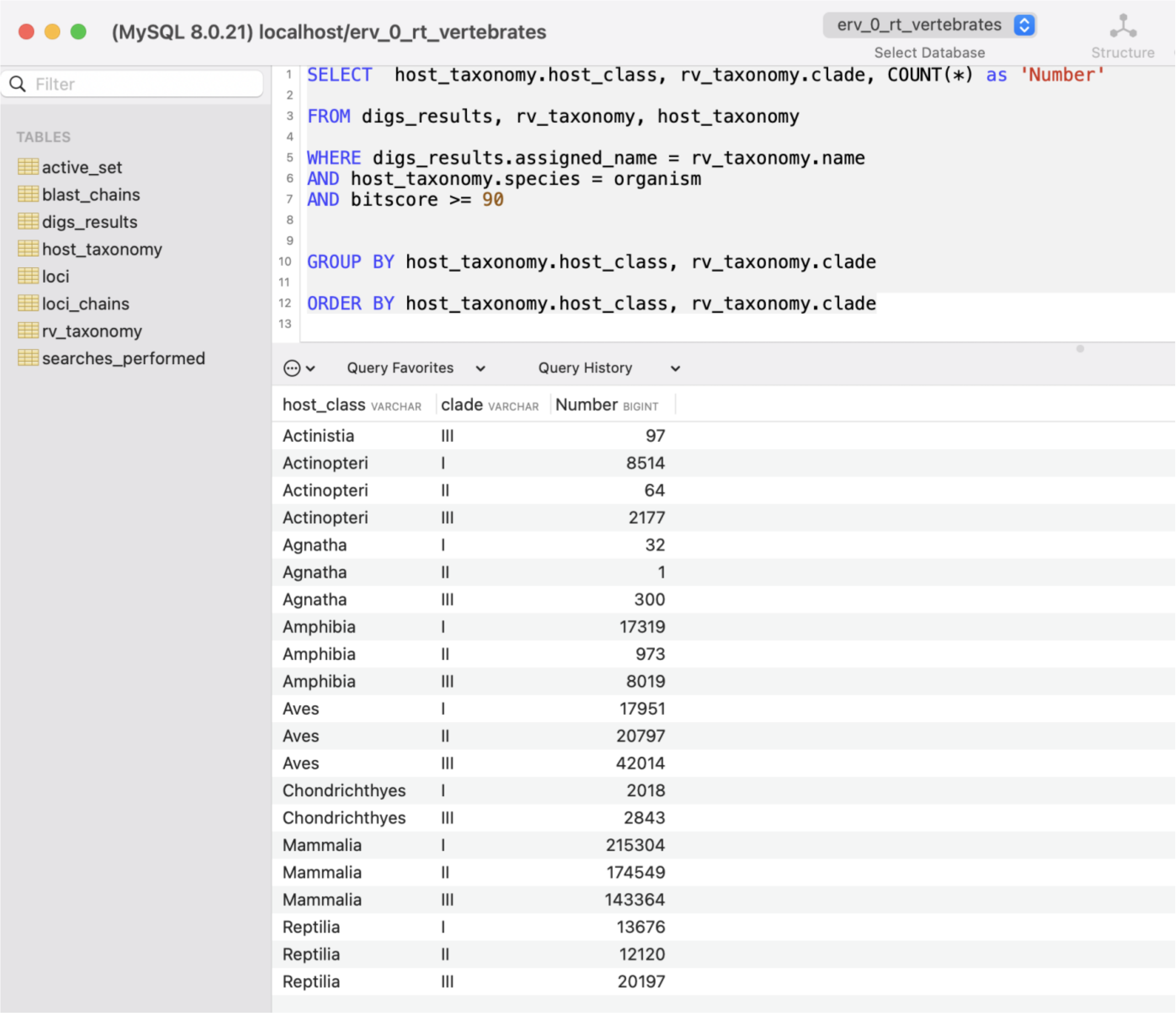


```sql
SELECT  host_taxonomy.host_class, rv_taxonomy.clade, COUNT(*) as 'Number'

FROM digs_results, rv_taxonomy, host_taxonomy

WHERE digs_results.assigned_name = rv_taxonomy.name
AND host_taxonomy.species = organism
AND bitscore >= 90


GROUP BY host_taxonomy.host_class, rv_taxonomy.clade

ORDER BY host_taxonomy.host_class, rv_taxonomy.clade
```

host_class VARCHAR	clade VARCHAR	Number BIGINT
Actinistia	III	97
Actinopteri	I	8514
Actinopteri	II	64
Actinopteri	III	2177
Agnatha	I	32
Agnatha	II	1
Agnatha	III	300
Amphibia	I	17319
Amphibia	II	973
Amphibia	III	8019
Aves	I	17951
Aves	II	20797
Aves	III	42014
Chondrichthyes	I	2018
Chondrichthyes	III	2843
Mammalia	I	215304
Mammalia	II	174549
Mammalia	III	143364
Reptilia	I	13676
Reptilia	II	12120
Reptilia	III	20197

Figure S3. Examples of SQL-based querying of DIGS results.

Panels show screenshots of a MySQL client program (Sequel Ace) connected to a screening database generated using the database-integrated genome screening (DIGS) tool. Structured query language (SQL) can be used to interrogate and manipulate screening databases. Each panel shows a distinct SQL query (upper window) and its results (lower window).

(a) Deriving stratified counts of non-retroviral EVE loci. The query shown here uses a custom table ('virus taxonomy',) linked via the 'assigned name' field (see **Fig. S2a)**, to group hits according to virus family. A 'digs results' table field, 'bit score', is used to restrict results to higher confidence hits **-** it is derived from the 'reverse' BLAST-step, in which a sequence hit identified from a 'forward' BLAST (i.e. probe versus target database file) is compared to the reference sequence library (RSL). It thereby provides an index showing how similar individual hits are to sequences included in the RSL.

(b) Retrieving information about paramyxovirus-derived EVEs recovered via DIGS. The query shown here provides an overview of paramyxovirus EVEs, showing the vertebrate class in which they were identified, which paramyxovirus species and genes they disclose similarity to (based on comparison to RSL sequences), and fields that show the closeness of the match. It uses two custom tables: (i) 'virus taxonomy' and (ii) 'host taxonomy,' which is linked via the 'organism' field (see **Fig. S2**). A bitscore cut-off is used to filter hits, and an order statement is used to sort results by the taxonomic designations of host species.

(c) An SQL query used to retrieve individual counts of high confidence endogenous retrovirus (ERV) reverse transcriptase (RT). RT hits were recovered by screening a target database comprising whole genome sequence data of 874 vertebrate species. We empirically determined that a bit score of >=90 eliminates false positive RT hits. Host and virus taxonomy tables, linked as described above for non-retroviral viruses, are used to stratify results.
