# Supplementary material for: A novel approach to exploring the dark genome and its application to mapping of the vertebrate virus fossil record
Source: Genome Biol. 2024 May 13;25:120. doi: 10.1186/s13059-024-03258-y (PMC11089739; doi:10.1186/s13059-024-03258-y)
Supplement: Supplementary file 4 — Additional file 4: Figure S4. Validation of the DIGS tool. [file 13059_2024_3258_MOESM4_ESM.pdf]

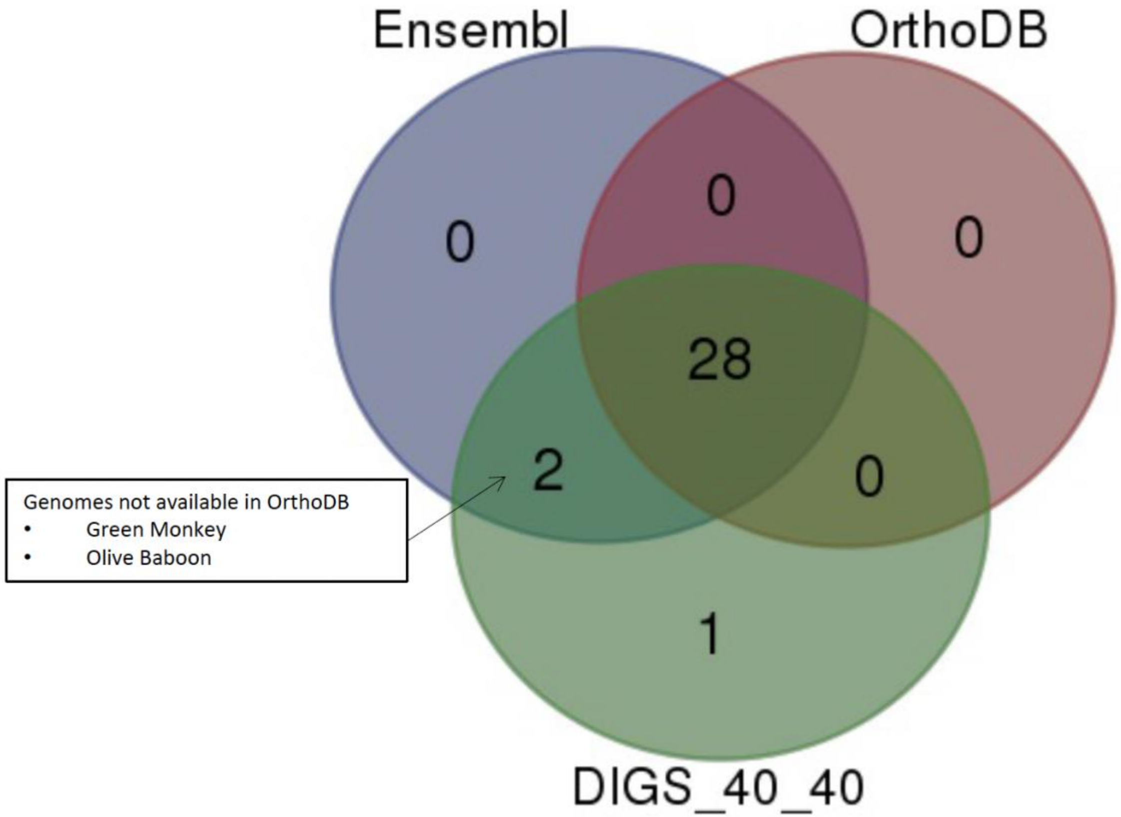

Figure S4. Validation of the DIGS tool.

A Venn diagram showing overlap among Tetherin/BST2 orthologs found using three different pipelines (OrthoDB, Ensembl, DIGS). For OrthoDB, Tetherin/BST2 orthologs were retrieved downloaded from the web service. Ensembl API version 79 was used to retrieve orthologs from Ensembl. The DIGS tool pipeline was run with bit score cutoffs of 40. Results from all three pipelines were compared based on the Ensembl Protein ID and were found to overlap by >99% as shown above.
