# Supplementary material for: A novel approach to exploring the dark genome and its application to mapping of the vertebrate virus fossil record
Source: Genome Biol. 2024 May 13;25:120. doi: 10.1186/s13059-024-03258-y (PMC11089739; doi:10.1186/s13059-024-03258-y)
Supplement: Supplementary file 5 — Additional file 5: Table S1. Putatively exogenous viruses identified in WGS data. [file 13059_2024_3258_MOESM5_ESM.pdf]

Table S1. Putatively exogenous viruses identified in WGS data

Vertebrate species[a]	Virus family/genus	Contig Accession #
**dsDNA**		
Bumblebee bat (*Craseonycteris thonglongyai*	*Adenoviridae**	PVKE010106839.1, PVKE010087958.1
Gracile opossum (*Gracilinanus agilis)*	*Adenoviridae**	JADWME010000011.1
Viscacha rat (*Octomys mimax*)		NDGM010762229.1, NDGM010239277.1, NDGM010144971.1, NDGM010804184.1
Ord's kangaroo rat (*Dipodomys ordii)*	*Adenoviridae**	n/a
Greater bamboo lemur (*Prolemur simus*)	*Papillomaviridae**	MPIZ01127437.1
Humpbacked dolphin (*Sousa chinensis*)	*Papillomaviridae**	QWLN01062956.1, QWLN01040988.1
Duck-billed platypus (*Ornithorhynchus anatinus*)	*Papillomaviridae*	n/a
Budgerigar (*Melopsittacus undulatus*)	*Herpesviridae*	JH541059
**ssDNA**		
David's myotis (*Myotis davidii*)	*Chaphamaparvovirus*	KB106247.1
White-faced capuchin (*Cebus imitator*)	*Chaphamaparvovirus*	KV391748.1
Brown mesite (*Mesitornis unicolor*)	*Chaphamaparvovirus*	n/a
Pit viper (*Protobothrops mucrosquamatus*)	*Chaphamaparvovirus*	n/a
David's myotis (*Myotis davidii*)	*Chaphamaparvovirus*	n/a
Canary (*Serinus canaria*)	*Chaphamaparvovirus*	n/a
Gulf pipefish	*Ichthamaparvovirus*	n/a
Senegalese bichir	*Protoparvovirus*-like	XM_039761881, XM_039762195
**ssRNA+ve**		
Vicugna pacos	*Hepacivirus*	ABRR02259018

Legend: [a] Common name (Latin binomial). * Previously unreported sequences, possibly derived from novel virus species. n/a = not available.
