# Supplementary material for: A novel approach to exploring the dark genome and its application to mapping of the vertebrate virus fossil record
Source: Genome Biol. 2024 May 13;25:120. doi: 10.1186/s13059-024-03258-y (PMC11089739; doi:10.1186/s13059-024-03258-y)
Supplement: Supplementary file 6 — Additional file 6: Figure S5. Genomic analysis of a superficially caulimovirus-like EVE. [file 13059_2024_3258_MOESM6_ESM.pdf]

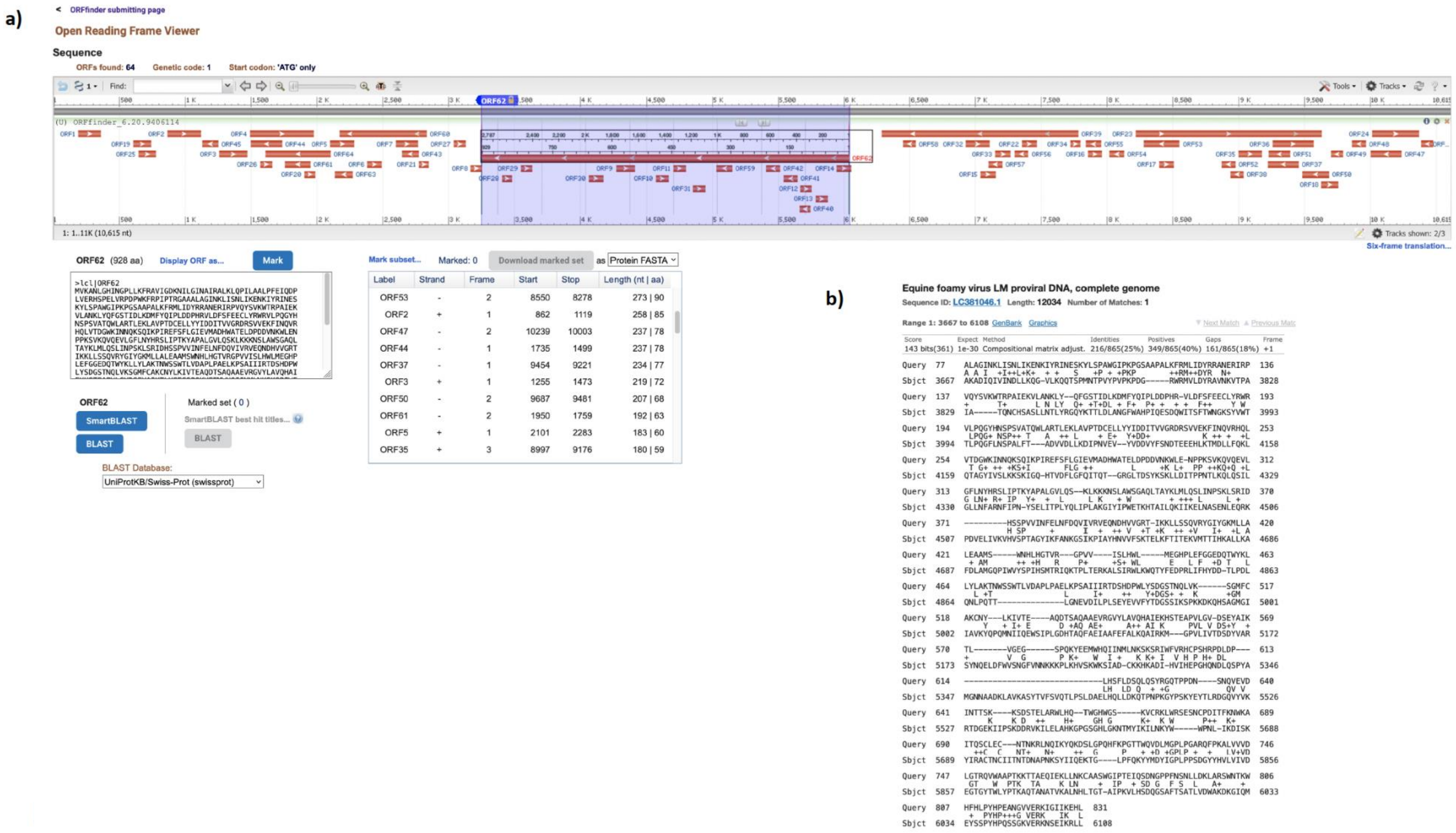

Figure S5. Genomic analysis of an endogenous retrovirus (ERV) sequence in the genome of the pig-nosed turtle (*Carettochelys insculpta*) that disclosed superficial similarity to caulimoviruses. (a) Predicted ORFs in the region of the EVE locus, determined using

NCBI’s ‘ORF finder’ program {Wheeler, 2003 #262}. The sequence input to ORF-finder included 5 kilobases of DNA sequence flanking the EVE locus. **(b)** tBLASTn result showing similarity of largest ORF to equine foamy virus.

References:

Wheeler, D.L., et al., *Database resources of the National Center for Biotechnology.* Nucleic Acids Res, 2003. **31**(1): p. 28-33.
