# Supplementary material for: A novel approach to exploring the dark genome and its application to mapping of the vertebrate virus fossil record
Source: Genome Biol. 2024 May 13;25:120. doi: 10.1186/s13059-024-03258-y (PMC11089739; doi:10.1186/s13059-024-03258-y)
Supplement: Supplementary file 7 — Additional file 7: Figure S6. Summary of vertebrate EVE coding potential. [file 13059_2024_3258_MOESM7_ESM.pdf]

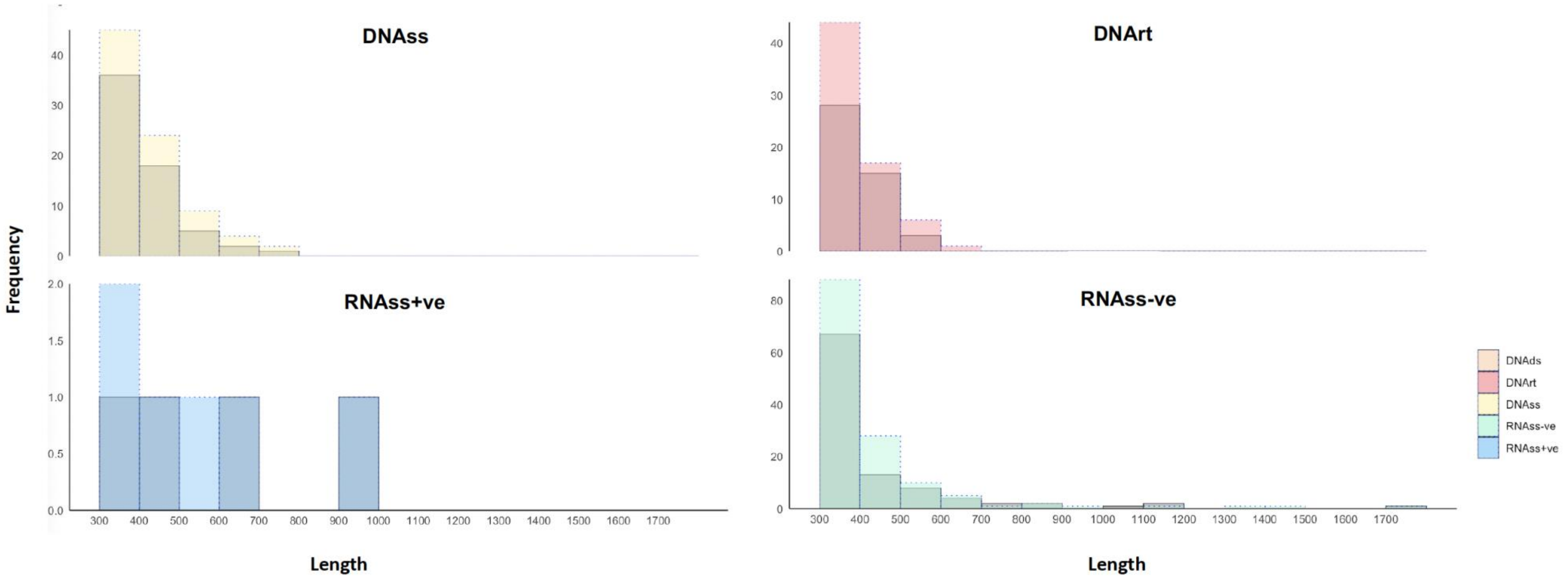

Figure S6. Summary of vertebrate EVE coding potential.

Histograms showing the distribution of lengths, binned in increments of 100 amino acid (aa) residues, of uninterrupted coding regions (lighter bars) and open reading frames (darker bars) longer than 300aa, contained within vertebrate endogenous viral elements (EVEs). was plotted in R using ggplot with ‘geom hist’ function.
