# Supplementary material for: A novel approach to exploring the dark genome and its application to mapping of the vertebrate virus fossil record
Source: Genome Biol. 2024 May 13;25:120. doi: 10.1186/s13059-024-03258-y (PMC11089739; doi:10.1186/s13059-024-03258-y)
Supplement: Supplementary file 8 — Additional file 8: Figure S7. Evolutionary relationships of vertebrate EVEs and viruses. [file 13059_2024_3258_MOESM8_ESM.pdf]

Herpesvirus *terminase*

a)

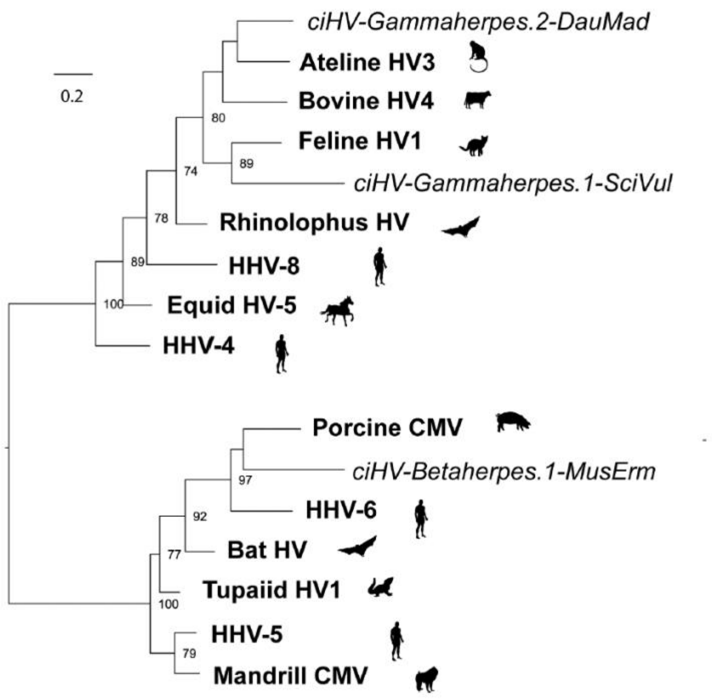

Herpesvirus *glycoprotein B*

b)

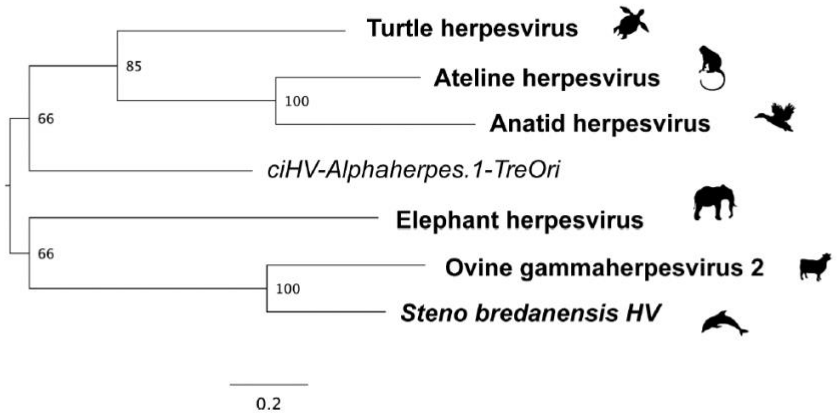

Alloherpesvirus *terminase*

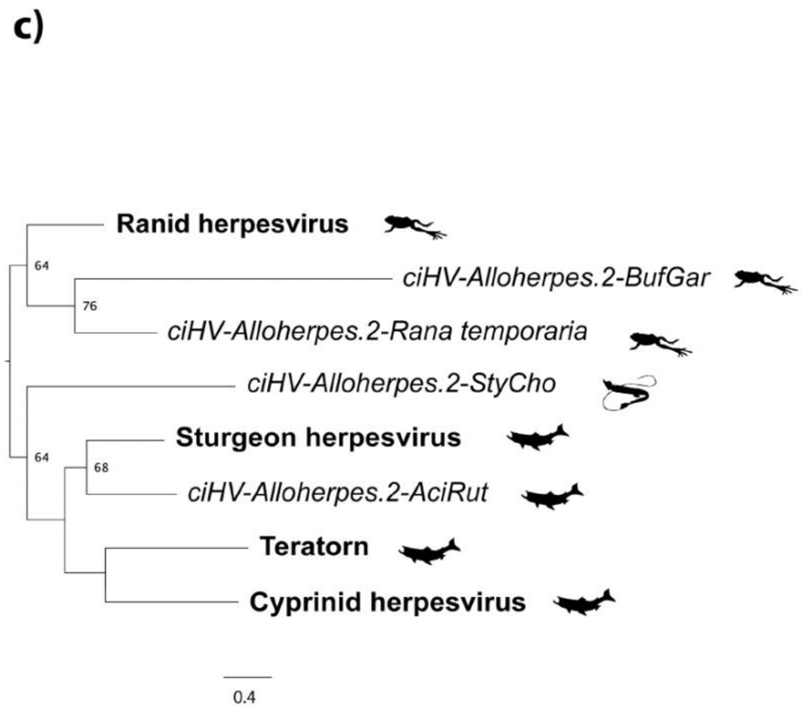

Circovirus *rep*

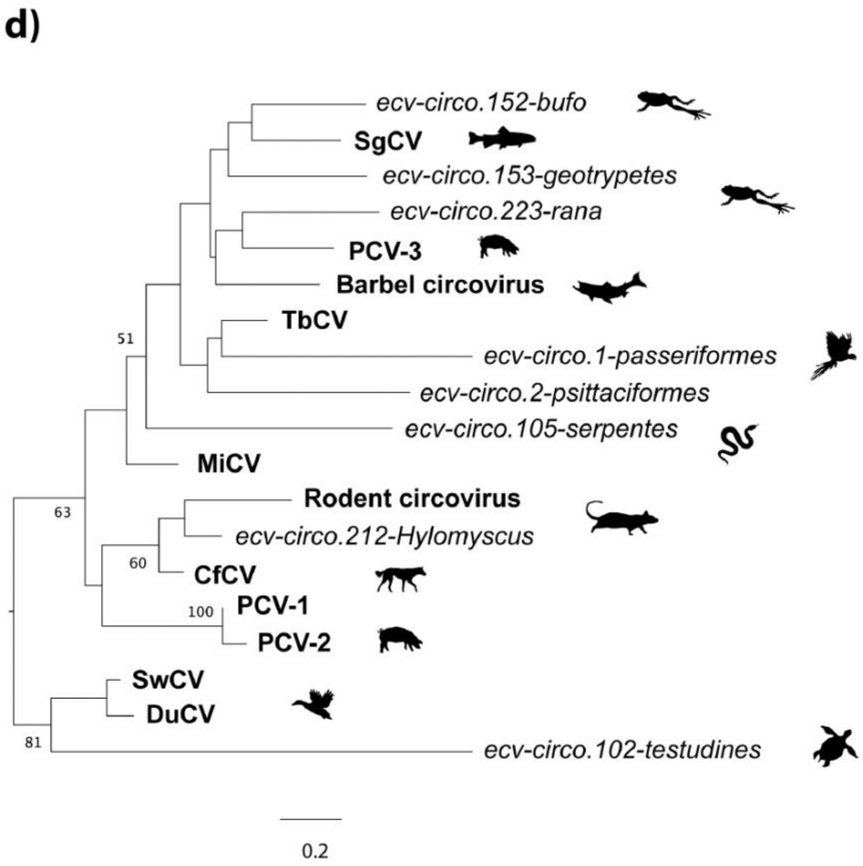

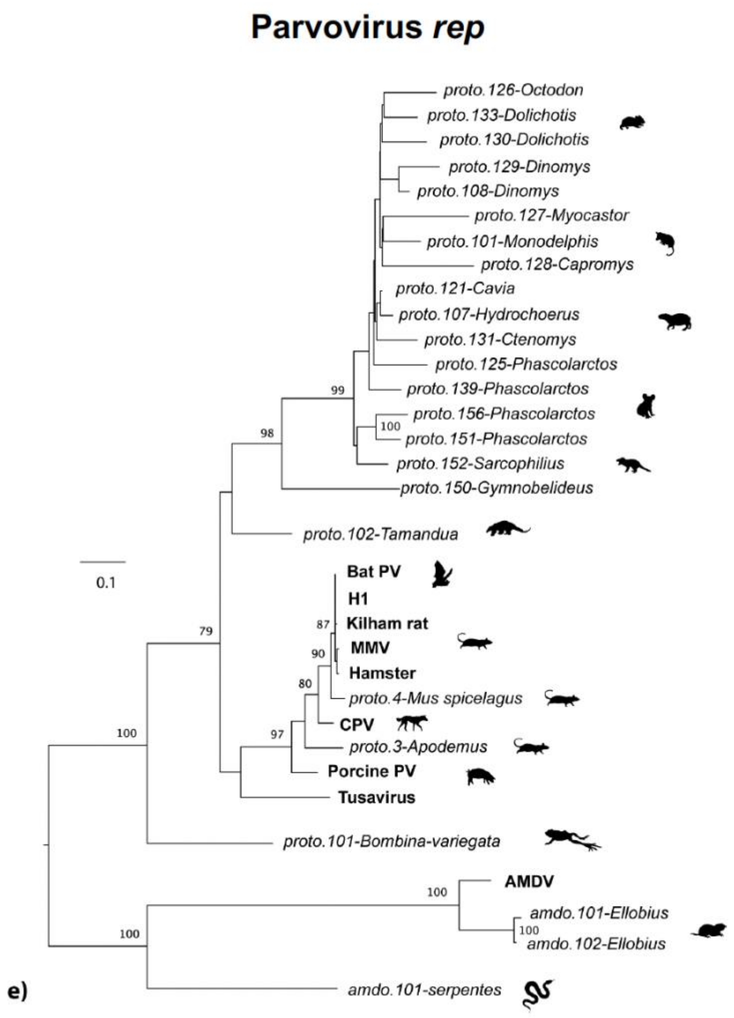
Parvovirus rep
proto.126-Octodon
proto.133-Dolichotis
proto.130-Dolichotis
proto.129-Dinomys
proto.108-Dinomys
proto.127-Myocastor
proto.101-Monodelphis
proto.128-Capromys
proto.121-Cavia
proto.107-Hydrochoerus
proto.131-Ctenomys
proto.125-Phascolarctos
proto.139-Phascolarctos
proto.156-Phascolarctos
proto.151-Phascolarctos
proto.152-Sarcophilius
proto.150-Gymnobelideus
proto.102-Tamandua
0.1
Bat PV
H1
Kilham rat
MMV
Hamster
proto.4-Mus spicelagus
CPV
proto.3-Apodemus
Porcine PV
Tusavirus
proto.101-Bombina-variegata
AMDV
amdo.101-Ellobius
amdo.102-Ellobius
amdo.101-serpentes
99
98
100
87
90
80
79
97
100
100
100
100
e)

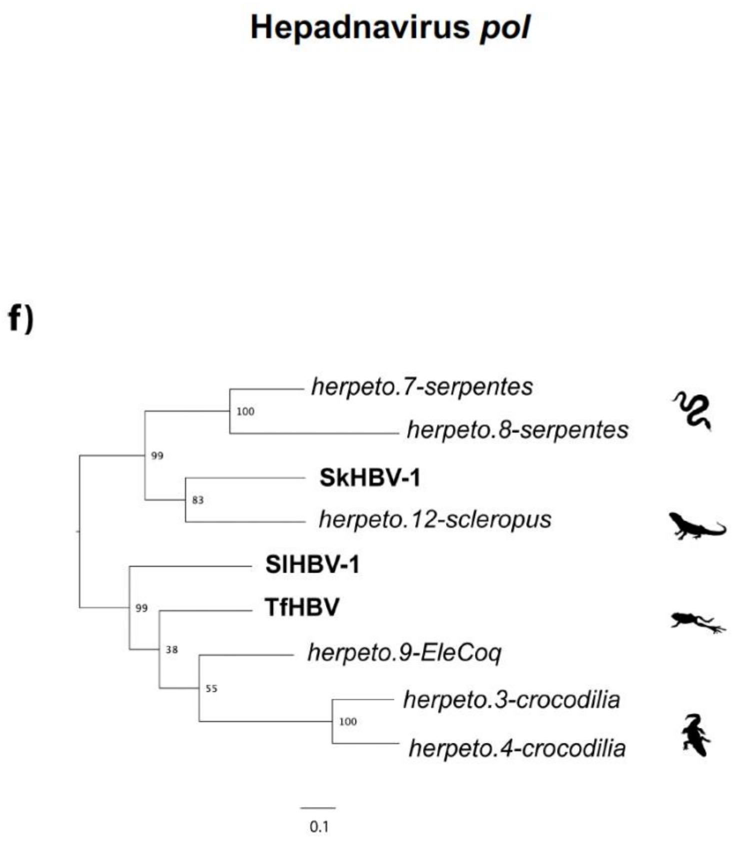
Hepadnavirus pol
f)
herpeto.7-serpentes
herpeto.8-serpentes
SkHBV-1
herpeto.12-scleropus
SlHBV-1
TfHBV
herpeto.9-EleCoq
herpeto.3-crocodilia
herpeto.4-crocodilia
100
99
83
99
38
55
100
0.1

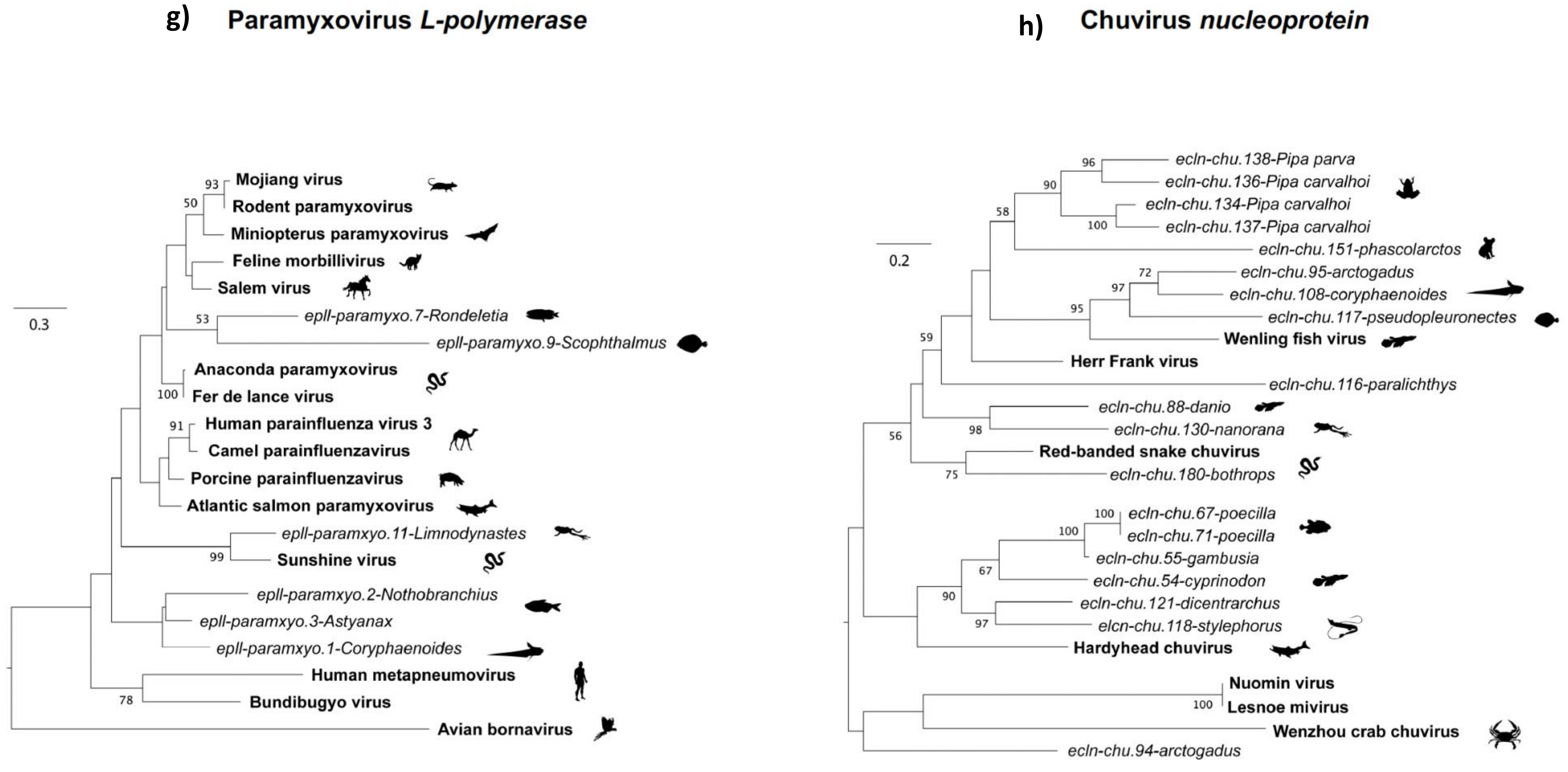

g) Paramyxovirus L-polymerase
h) Chuvirus nucleoprotein
0.3
Mojiang virus
Rodent paramyxovirus
Miniopterus paramyxovirus
Feline morbillivirus
Salem virus
epll-paramyxo.7-Rondeletia
epll-paramyxo.9-Scophthalmus
Anaconda paramyxovirus
Fer de lance virus
Human parainfluenza virus 3
Camel parainfluenzavirus
Porcine parainfluenzavirus
Atlantic salmon paramyxovirus
epll-paramxyo.11-Limnodynastes
Sunshine virus
epll-paramxyo.2-Nothobranchius
epll-paramxyo.3-Astyanax
epll-paramxyo.1-Coryphaenoides
Human metapneumovirus
Bundibugyo virus
Avian bornavirus
93
50
53
100
91
99
78
0.2
ecln-chu.138-Pipa parva
ecln-chu.136-Pipa carvalhoi
ecln-chu.134-Pipa carvalhoi
ecln-chu.137-Pipa carvalhoi
ecln-chu.151-phascolarctos
ecln-chu.95-arctogadus
ecln-chu.108-coryphaenoides
ecln-chu.117-pseudopleuronectes
Wenling fish virus
Herr Frank virus
ecln-chu.116-paralichthys
ecln-chu.88-danio
ecln-chu.130-nanorana
Red-banded snake chuvirus
ecln-chu.180-bothrops
ecln-chu.67-poecilla
ecln-chu.71-poecilla
ecln-chu.55-gambusia
ecln-chu.54-cyprinodon
ecln-chu.121-dicentrarchus
elcn-chu.118-stylephorus
Hardyhead chuvirus
Nuomin virus
Lesnoe mivirus
Wenzhou crab chuvirus
ecln-chu.94-arctogadus
96
90
100
58
72
97
95
59
98
56
75
100
100
67
90
97
100

i) **Bornavirus *L-polymerase***

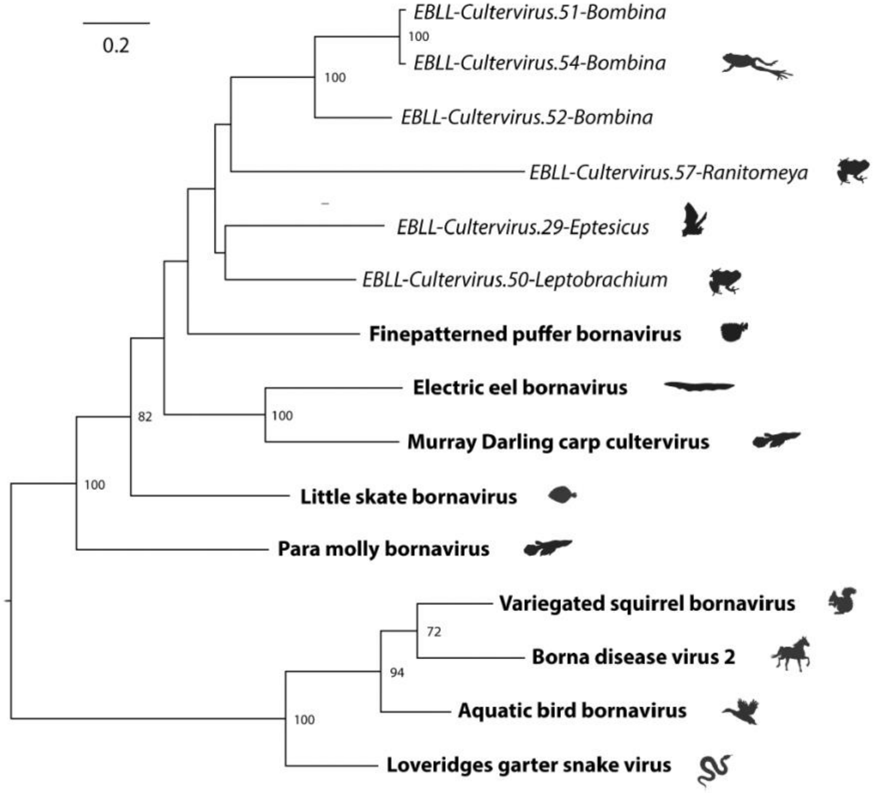

j) **Bornavirus *glycoprotein***

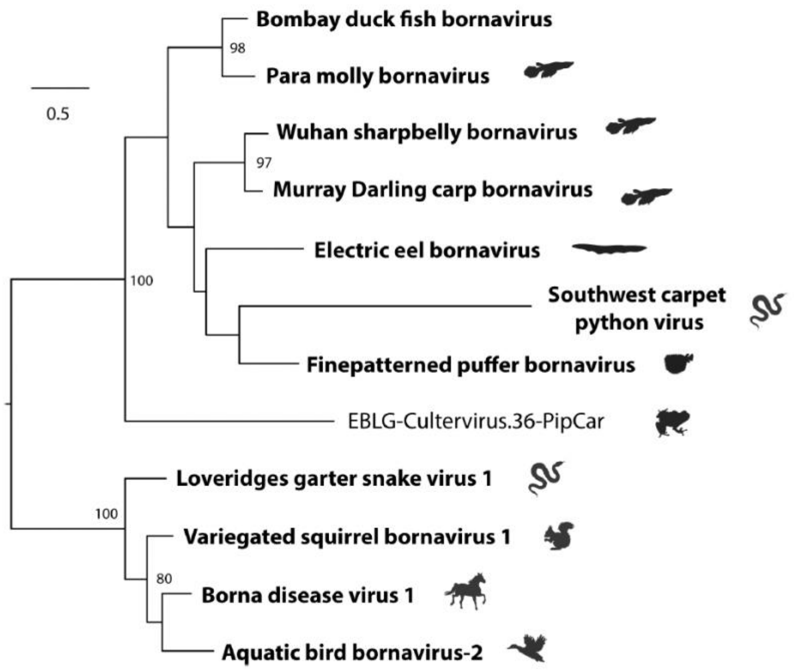

Figure S7. Evolutionary relationships of vertebrate EVEs and viruses.
Bootstrapped maximum likelihood trees showing the reconstructed evolutionary relationships between vertebrate EVEs and related viruses. **(a)** Gammaherpesvirus *terminase*; **(b)** Alphaherpesvirus *glycoprotein B*; **(c)** Alloherpesvirus *terminase*; **(d)** Circovirus *rep*; **(e)** Parvovirus *rep*; **(f)** Hepadnavirus *pol*; **(g)** Paramyxovirus *L polymerase*; **(h)** Chuvirus *nucleoprotein*; **(i)** Bornavirus *L polymerase*; **(j)** Bornavirus *glycoprotein*. Numbers on nodes indicate bootstrap

support (100 bootstrap replicates). All trees were constructed from nucleotide level alignments using the General Time Reversible (GTR) model of nucleotide substitution. Scale bars show evolutionary distance in substitutions per site. Taxon names are shown in bold for viruses, italics for EVEs. Where EVE sequences occurred in >1 host species (indicated by a host group rather than a species name in the EVE identifier), consensus sequences were analysed. Where EVE sequences were identified in a single species, the species name is shown using a three letter abbreviation (e.g. 'DauMad' for *Daubentonia madagascariensis*).
