# Supplementary material for: A novel approach to exploring the dark genome and its application to mapping of the vertebrate virus fossil record
Source: Genome Biol. 2024 May 13;25:120. doi: 10.1186/s13059-024-03258-y (PMC11089739; doi:10.1186/s13059-024-03258-y)
Supplement: Supplementary file 9 — Additional file 9: Figure S8. Amplified lineages of endogenous viral elements. [file 13059_2024_3258_MOESM9_ESM.pdf]

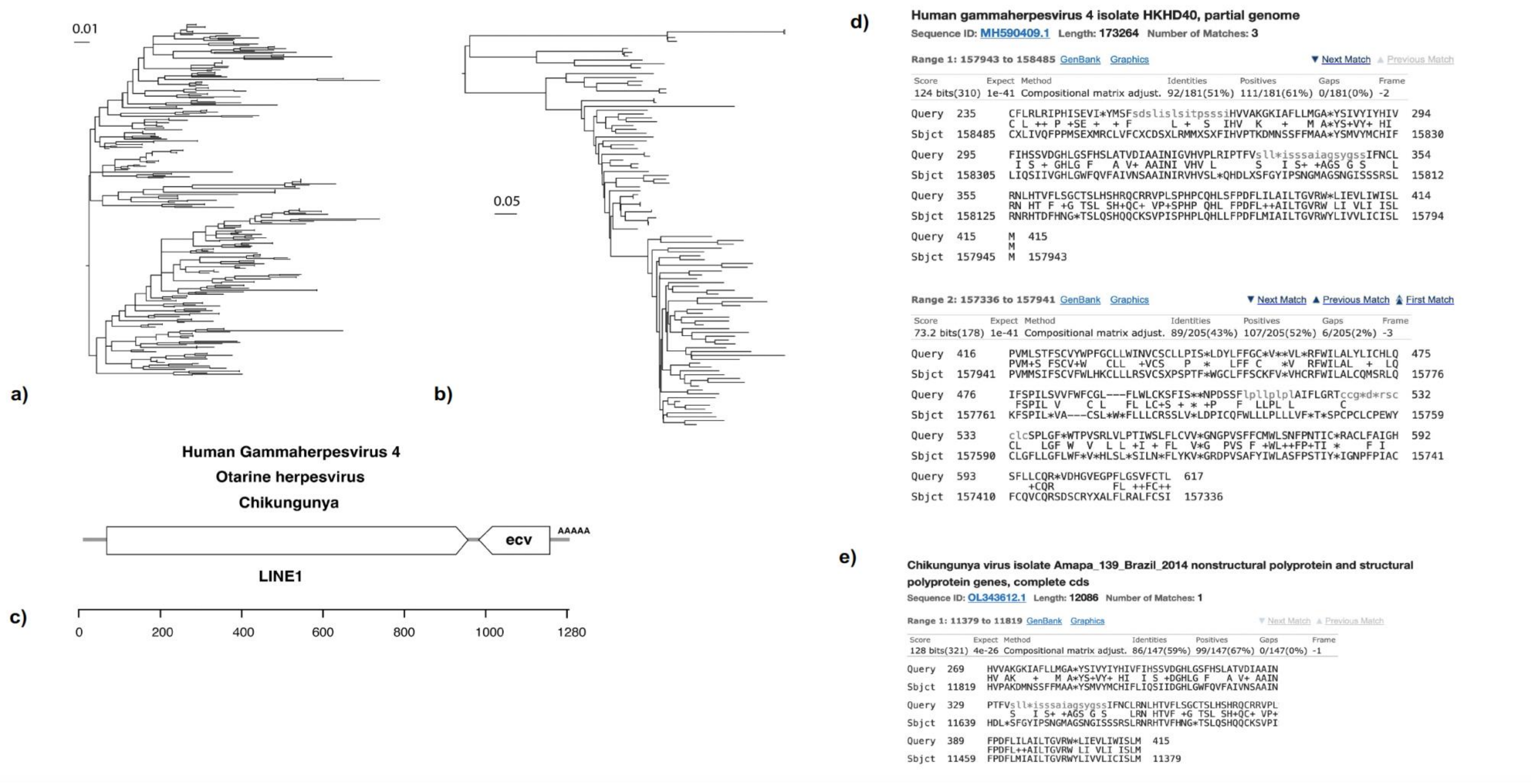


**Figure S8. Amplified lineages of endogenous viral elements.**

**Panels (a-b)** Maximum likelihood (ML) phylogenies showing reconstructed evolutionary relationships among **(a)** an lineage of endogenous hepatitis B (eHBV) elements, labelled ‘ehbv-avi.27-Suliformes’, that has been amplified in cormorant (family Phalacrocoracidae) genomes and; **(b)** a lineage of long interspersed

nuclear element (LINE)-associated endogenous circoviral elements (ECVs), labelled ‘ecv-circo.51-Carnivora’, amplified in carnivore genomes (order Carnivora). ML phylogenies were reconstructed from nucleotide-level alignments. Scale bars show evolutionary distance in substitutions per site. **(c)** Genomic structure found in an ecv-circo.51-Carnivora subclade, comprised of a LINE1 homologous region (left) and region homologous to a circoviral *rep* gene (right). The orientation of each of these sub-components is indicated, and the presence of a poly-adenine (A) tail. Homology of the LINE1 region to human gammaherpesvirus 4 and an isolate of Chikungunya virus is indicated in panel **(d)** and **(e)**, respectively.
