# Supplementary material for: A novel approach to exploring the dark genome and its application to mapping of the vertebrate virus fossil record
Source: Genome Biol. 2024 May 13;25:120. doi: 10.1186/s13059-024-03258-y (PMC11089739; doi:10.1186/s13059-024-03258-y)
Supplement: Supplementary file 11 — Additional file 11: Figure S9. Germline incorporation through time shown separately for each virus family. [file 13059_2024_3258_MOESM11_ESM.pdf]

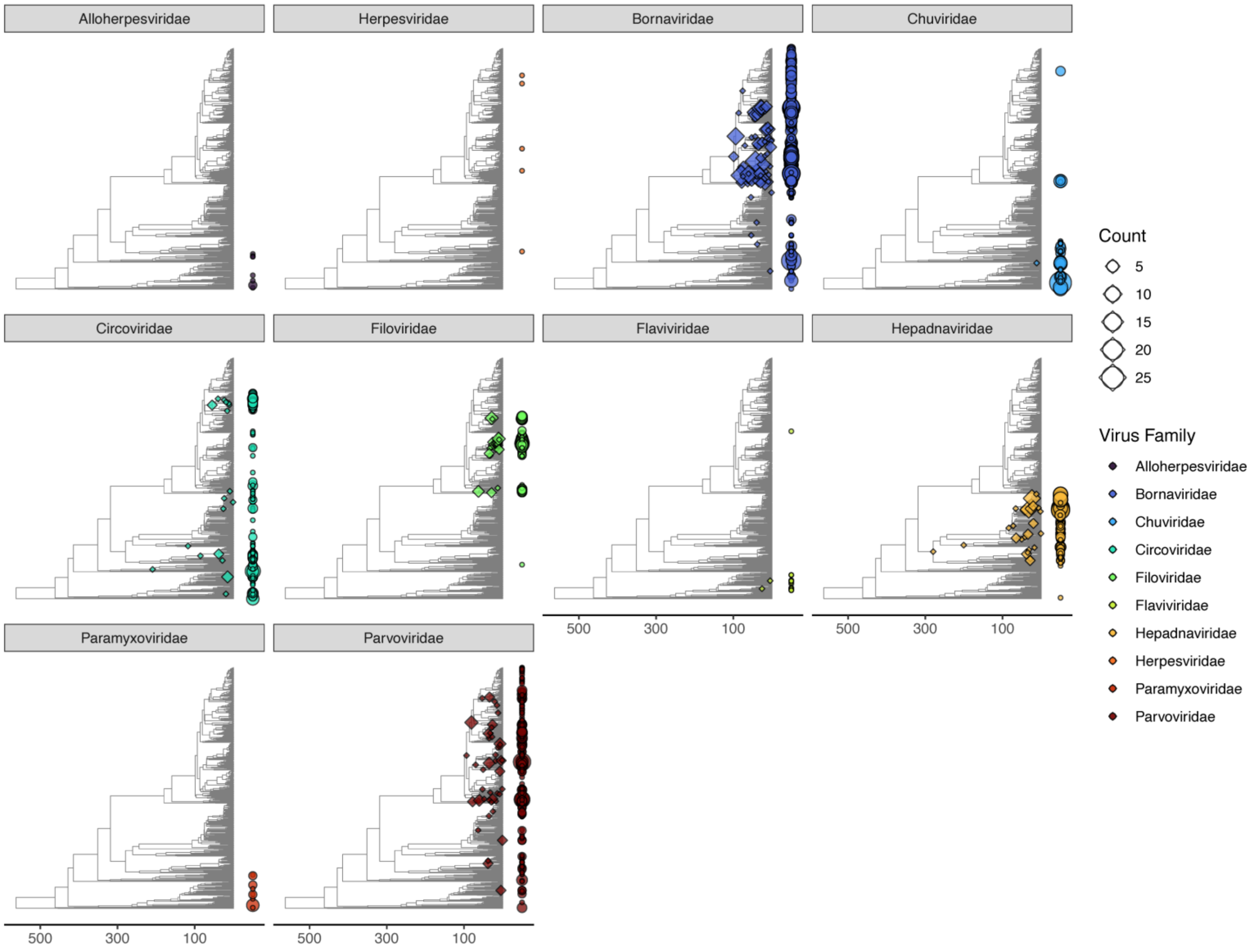

Figure S9. Time-calibrated phylogenies of vertebrate species examined in this study, obtained via TimeTree [119]. Minimum ages of endogenization events are indicated by diamonds on internal nodes for EVE loci present as orthologs in multiple species. The distribution of 10 families of viruses is shown across vertebrates separately. The presence of EVE sequences in each species

genome is indicated by circles at phylogeny tips. Circles and diamonds nodes are scaled by the number of sequences detected and color-coded by virus family as indicated in legend. For circles, scaling indicates the total number of EVE sequences detected within each species genome, including both unique and shared endogenization events.
