# Supplementary material for: A novel approach to exploring the dark genome and its application to mapping of the vertebrate virus fossil record
Source: Genome Biol. 2024 May 13;25:120. doi: 10.1186/s13059-024-03258-y (PMC11089739; doi:10.1186/s13059-024-03258-y)
Supplement: Supplementary file 12 — Additional file 12. Review history. [file 13059_2024_3258_MOESM12_ESM.docx]

Review History

**First round of review**

**Reviewer 1**

**Are you able to assess all statistics in the manuscript, including the appropriateness of statistical tests used?** There are no statistics in the manuscript.

**Comments to author:**

The manuscript by Blanco-Melo et al provides an tool (DIGS) to study endogenous viral elements (EVEs). The manuscript presents an impressive set of EVEs, some found earlier and some new ones, among the vertebrate genomes studied. The findings are highly original and scientifically very relevant, the tool is useful for future studies, and - although there is a great amount of data - the writing of the manuscript is excellent with data presented in a very clear way. Below a few suggestions for improvement, which are typos only.

- Page 13, line 27. "with robustly" can be deleted

- Page 18, line 13. "to" is missing

- Page 26, line 10. A reference is missing

**Reviewer 2**

**Are you able to assess all statistics in the manuscript, including the appropriateness of statistical tests used?** No, I do not feel adequately qualified to assess the statistics.

**Comments to author:**

In their manuscript, Blanco-Melo and colleagues present DIGS, a sequence similarity-based screening pipeline linked to a relational database management system. Using DIGS, the authors identify >1 million sequences of endogenous viral elements (EVEs) in 874 vertebrate genomes. As expected, the vast majority of these EVEs represent endogenous retroviruses (ERVs). However, the authors also identified more than 6000 sequences representing other virus families, including borna-, filo- and flaviviruses. While some of these sequences had previously been identified as EVEs (and others may be the result of WGS contamination), Blanco-Melo and colleagues also identify several new EVEs. For example, they provide evidence for amphibian filoviruses by identifying a filovirus-like sequence in a frog. Furthermore, their findings suggest the long-term existence of two distinct filovirus lineages infecting mammals.

Although similar searches for EVEs have been performed before and although there is some overlap with previous studies, the present screening approach is clearly more comprehensive and complete. Furthermore, all parts of the manuscripts are beautifully written and it was a pleasure to read. I would also like to highlight the high quality of the figures, most of which are well-designed and well thought out.

I am listing a few points below that may help to further improve the quality of the manuscript.

Major points:

(1) The authors discuss the possibility of contamination by DNA derived from exogenous (latent/chronic) viruses. Furthermore, the identification of plant viruses such as geminiviruses strongly suggests that some hits may be the result of diet-related contamination. In my opinion, potential sources of contamination and false positive matches may still be discussed in more detail. While plant viruses may be easily identified as diet-related contamination in samples from herbivores, it may be more difficult to identify diet contaminations in carnivore samples, especially since animals can ingest both exogenous and endogenous viruses via their diet.

(2) The authors exclude "EVEs derived from DNAds viruses" (page 17, lines 15/16) when determining the frequency of germline incorporation events. Still, DNAds are included in Fig. 6. Why?

Minor points:

(1) Page 9, line 1 and Fig. 3: Doesn't "Squamata" describe an order, while "Reptilia" is the class? Are Reptilia or Squamata shown in Fig. 3?

(2) Figure 3 shows one class II element for Agnatha, although page 9, line 15 highlights that this class is "completely absent from agnathans". It only gets clear later (Table 2) that this single class II ERV may actually be a contamination. Would it make sense to omit likely contaminants from Fig. 3? Alternatively, the authors may highlight them in a different color/shape.

(3) Page 15: amphibians may also be listed in line 9, since Fig. 4 suggests that they also harbor Chuvirus-like sequences.

(4) Page 26, line 10: "[ref]" needs to be replaced.

(5) The legend and description of Figure S4 may be improved. What exactly is indicated by the numbers in the Venn diagram? What kind of overlap was determined?

(6) Page 20, line 26 mentions "700 000 high confidence" ERV matches, while "a high confidence set of 500,701 loci" was identified according to page 8. Which number is correct?

(7) Although the snake filovirus TAPV is mentioned several times, Fig. 4 implies absence of filoviruses from snakes.

(8) Fig. 4: To my knowledge, Sphenodontia are a subgroup of Lepidosauria. Thus, all the viruses found in Sphenodontia should also be present in Lepidosauria (incl. Metahepadnaviruses).

(9) Typos:

- Legend of Figure S3(a): "see Fig. S"

- Legend of figure 2, page 34, lines 16/18: "(f)" is used twice

- Page 13 line 27: "element groups with robustly within a clade"

**Reviewer 3**

**Are you able to assess all statistics in the manuscript, including the appropriateness of statistical tests used?** Yes, and I have assessed the statistics in my report.

**Comments to author:**

The sequencing of genomes is progressing rapidly, but deciphering the complex layers of information they contain is a real challenge. In the era of technological progress that is producing whole genome sequencing (WGS), new analytical tools are needed. The whole genome sequences are generally comprised of genomic "dark matter". The manuscript is about a novel approach called database-integrated genome screening (DIGS) that aims to explore this "dark matter" which refers to genomic regions that are poorly understood. The focus of the study is on endogenous viral elements (EVEs) found within vertebrate genomes, which can impact host biology and serve as a "fossil record" of viruses.

While several previous studies have described endogenous viral element (EVE) diversity in vertebrates, the current study is significantly more extensive and comprehensive. In addition, the strategy also includes analyses for non-retroviral viruses, which represents a new level of investigation. It is foreseeable that it will be a widely used analysis tool.

The method used to identify endogenous viral elements (EVEs) in the study is called Database-Integrated Genome Screening (DIGS). This approach involves linking sequence similarity searches with a relational database. By using sequence similarity searches, such as the Basic Local Alignment Search Tool (BLAST), researchers can identify novel genome features based on their resemblance to previously described ones. This approach, called DIGS (Database-Integrated Genome Screening) provides an accessible platform for a wide range of genomics investigations. The authors also describe an open software framework for performing DIGS. The manuscript emphasizes the importance of providing detailed descriptions of resources used in the Methods section, including the use of Research Resource Identifiers (RRIDs) where possible.

There are clear advantages of the proposed DIGS approach:

a. Comprehensive survey: The proposed DIGS strategy allows for a comprehensive survey of EVE diversity across a broad range of vertebrate species, providing a panoramic perspective on vertebrate virus evolution.

b. Among the strengths is the inclusion of a phylogenetic level of analysis, which is useful for distinguishing between a true EVE event and contamination.

c. Accessibility: The DIGS approach provides an accessible platform for a wide range of genomics investigations, allowing researchers to explore the "dark genome" in silico.

d. Open software framework: The study also describes an open software framework for performing DIGS, making it easier for other researchers to replicate and build upon the findings.

The limitations include:

a. Reliance on sequence similarity: The DIGS approach relies on sequence similarity searches, which may not always accurately identify novel genome features. There is a possibility of false positive or false negative results.

b. Limited to known sequences: The DIGS approach is limited to identifying EVEs that have some resemblance to previously described sequences. It may miss entirely novel or highly divergent EVEs that do not show significant sequence similarity to known ones.

c. Size limitation.

d. The approach may be limited for identifying recently integrated EVEs. How does the approach work for recently integrated viral sequences in primates?

Overall, this article discusses the power of linking a relational database management system to a similarity search-based screening pipeline for in silico exploration of the dark genome. The DIGS approach used in the study provides a valuable tool for exploring the dark genome and identifying EVEs, but it has its limitations in terms of relying on sequence similarity and potentially missing novel or highly divergent EVEs.

The questions asked in this study include

(i) How many EVE sequences were identified using the database-integrated genome screening (DIGS) approach?

Using the DIGS approach, developed by the authors, approximately 1.1 million endogenous viral element (EVE) sequences were identified.

(ii) Were there any previously unreported EVEs discovered in the study? The study identified a set of clearly novel endogenous viral elements (EVEs) that had not been reported before. These novel EVEs were defined as either deriving from a virus group not previously reported in the host group or occurring in species distantly related to those in which similar EVEs had been identified previously. The study included these novel EVEs in their analysis, expanding our knowledge of virus diversity and distribution among vertebrate hosts.

(iii) What are the main types of EVEs found in vertebrate genomes?

Addressing this question, the study raises several interesting points, and some concerns:

- The DIGS approach could be useful to identify EVEs that are conserved in all vertebrate classes - would be worth a paragraph in the discussion.

- Page 12, top: "We also identified and excluded hits that appeared likely to derived from exogenous viruses …". I might have missed, but would be important to have more details on how the Figure 4 was generated? How was external viruses and EVEs were distinguished?

- Figure 4 has some contradiction with the main text: Page 26: "The absence, or near absence, of many virus groups from our catalogue of vertebrate EVEs is noteworthy. For example, vertebrates are infected with many ssRNA+ve viruses, but EVEs derived from these viruses are extremely rare,…" By contrast, Figure 4 suggests that ssRNA+ve viruses are integrated in mammals and generate EVEs. Flaviviruses are highly interesting, because they are ssRNA+ve viruses, similarly to SARS-Cov viruses, and there has been a recent debate on this very topic - see DOI: 10.1126/science.372.6543.674.

- Figure 4 - There seems to be some contradiction also on DNArt, reverse-transcribing DNA viruses. Figure 4 suggest Hepadnaviridae EVE are present in mammals, the main text does not discuss it, and Figure 6 does not seem to support it - no significant germline incorporation rate in mammals.

- Page 22: "Additionally, uncharacterised EVE loci may be hard to recognise due to their being

highly degraded or fragmented, or because their exogenous virus counterparts are either

unknown or extinct [65, 66]." All EVEs are fragmented or there are others that are better preserved?

- Page 10: "Furthermore, the initial integrated form of a retrovirus genome, called a pro-virus, is typically replication competent and increases in germline copy number can thus occur through reinfection of germ line cells [32]. " To be more precise. Endogenous retrovi-ruses (ERVs) have an evolutionary life cycle when they function as transposable elements and can increase the copy number of the germline without leaving the germline and re-infecting.

Minor:

Page 18: "Notably, within the Mammal-1 group, EVEs identified in mammals indigenous to Southern Hemisphere continents (e.g. marsupials, xenarthrans) group …"

Evolutionary biologist would argue that Marsupial are not mammals.

Page 28: "DIGS has previously been used to broadly survey the distribution of interferon

stimulated genes in mammals [ref],…" - The reference is missing

Figure 4 - There are no abbreviations on the figure. The abbreviations can only be found on Figure 6, but it would be important to add them here as well.

**Authors Response**

**Point-by-point responses to the reviewers’ comments:**

Reviewer 1

The manuscript by Blanco-Melo et al provides an tool (DIGS) to study endogenous viral elements (EVEs). The manuscript presents an impressive set of EVEs, some found earlier and some new ones, among the vertebrate genomes studied. The findings are highly original and scientifically very relevant, the tool is useful for future studies, and - although there is a great amount of data - the writing of the manuscript is excellent with data presented in a very clear way. Below a few suggestions for improvement, which are typos only.

***Thank you!***

- Page 13, line 27. "with robustly" can be deleted

***Change made.***

- Page 18, line 13. "to" is missing

***Change made.***

- Page 26, line 10. A reference is missing.

***Thank you for spotting this, we have included the missing reference in the updated manuscript.***

Reviewer 2

In their manuscript, Blanco-Melo and colleagues present DIGS, a sequence similarity-based screening pipeline linked to a relational database management system. Using DIGS, the authors identify >1 million sequences of endogenous viral elements (EVEs) in 874 vertebrate genomes. As expected, the vast majority of these EVEs represent endogenous retroviruses (ERVs). However, the authors also identified more than 6000 sequences representing other virus families, including borna-, filo- and flaviviruses. While some of these sequences had previously been identified as EVEs (and others may be the result of WGS contamination), Blanco-Melo and colleagues also identify several new EVEs. For example, they provide evidence for amphibian filoviruses by identifying a filovirus-like sequence in a frog. Furthermore, their findings suggest the long-term existence of two distinct filovirus lineages infecting mammals.

Although similar searches for EVEs have been performed before and although there is some overlap with previous studies, the present screening approach is clearly more comprehensive and complete. Furthermore, all parts of the manuscripts are beautifully written and it was a pleasure to read. I would also like to highlight the high quality of the figures, most of which are well-designed and well thought out.

***Thank you!***

Major points:

(1) The authors discuss the possibility of contamination by DNA derived from exogenous (latent/chronic) viruses. Furthermore, the identification of plant viruses such as geminiviruses strongly suggests that some hits may be the result of diet-related contamination. In my opinion, potential sources of contamination and false positive matches may still be discussed in more detail. While plant viruses may be easily identified as diet-related contamination in samples from herbivores, it may be more difficult to identify diet contaminations in carnivore samples, especially since animals can ingest both exogenous and endogenous viruses via their diet.

***Thank you for this suggestion. We agree this is an interesting dimension of our study and have added some clarification to the results (p10 lines 5-18), and some discussion incorporating the points raised by the reviewer: p25: lines 7-19.***

(2) The authors exclude "EVEs derived from DNAds viruses" (page 17, lines 15/16) when determining the frequency of germline incorporation events. Still, DNAds are included in Fig. 6. Why?

**Thank you for spotting this oversight. We have updated the text to correctly read “We excluded EVEs derived from Polinton-like viruses (Adintoviruses) and teratorn elements, both of which exhibit relatively high copy numbers due to intragenomic amplification”. We removed the sentence following this, which referred to a early version of Figure 6.**

Minor points:

(3) Page 15: amphibians may also be listed in line 9, since Fig. 4 suggests that they also harbor Chuvirus-like sequences.

***Indeed they do. The text has been updated.***

(5) The legend and description of Figure S4 may be improved. What exactly is indicated by the numbers in the Venn diagram? What kind of overlap was determined?

***We apologise for this omission. The full legend for the figure has been included in the revised manuscript***

(6) Page 20, line 26 mentions "700 000 high confidence" ERV matches, while "a high confidence set of 500,701 loci" was identified according to page 8. Which number is correct?

***We thank the reviewer for their careful reading of the manuscript. We have now made sure the correct number (702,329) is referenced throughout the manuscript. The incorrect number reflected errors in Table 2 which have been corrected in the revised manuscript.***

(7) Although the snake filovirus TAPV is mentioned several times, Fig. 4 implies absence of filoviruses from snakes.

***Thank you for spotting this oversight! We only became aware of the existence of snake filoviruses (TABV) after making figure 4. We have updated the figure accordingly.***

(8) Fig. 4: To my knowledge, Sphenodontia are a subgroup of Lepidosauria. Thus, all the viruses found in Sphenodontia should also be present in Lepidosauria (incl. Metahepadnaviruses).

***Thank you for highlighting this error. We have corrected figure so that it displays the correct taxonomic groups within Lepidosauria (Squamata and Rhynchocephalia).***

(9) Typos:

- Legend of Figure S3(a): "see Fig. S"

***Thank you for spotting, we have updated the figure legend with the correct figure reference.***

Reviewer 3

The sequencing of genomes is progressing rapidly, but deciphering the complex layers of information they contain is a real challenge. In the era of technological progress that is producing whole genome sequencing (WGS), new analytical tools are needed. The whole genome sequences are generally comprised of genomic "dark matter". The manuscript is about a novel approach called database-integrated genome screening (DIGS) that aims to explore this "dark matter" which refers to genomic regions that are poorly understood. The focus of the study is on endogenous viral elements (EVEs) found within vertebrate genomes, which can impact host biology and serve as a "fossil record" of viruses.

While several previous studies have described endogenous viral element (EVE) diversity in vertebrates, the current study is significantly more extensive and comprehensive. In addition, the strategy also includes analyses for non-retroviral viruses, which represents a new level of investigation. It is foreseeable that it will be a widely used analysis tool.

The method used to identify endogenous viral elements (EVEs) in the study is called Database-Integrated Genome Screening (DIGS). This approach involves linking sequence similarity searches with a relational database. By using sequence similarity searches, such as the Basic Local Alignment Search Tool (BLAST), researchers can identify novel genome features based on their resemblance to previously described ones. This approach, called DIGS (Database-Integrated Genome Screening) provides an accessible platform for a wide range of genomics investigations. The authors also describe an open software framework for performing DIGS. The manuscript emphasizes the importance of providing detailed descriptions of resources used in the Methods section, including the use of Research Resource Identifiers (RRIDs) where possible.

There are clear advantages of the proposed DIGS approach:

a. Comprehensive survey: The proposed DIGS strategy allows for a comprehensive survey of EVE diversity across a broad range of vertebrate species, providing a panoramic perspective on vertebrate virus evolution.

b. Among the strengths is the inclusion of a phylogenetic level of analysis, which is useful for distinguishing between a true EVE event and contamination.

c. Accessibility: The DIGS approach provides an accessible platform for a wide range of genomics investigations, allowing researchers to explore the "dark genome" in silico.

d. Open software framework: The study also describes an open software framework for performing DIGS, making it easier for other researchers to replicate and build upon the findings.

The limitations include:

a. Reliance on sequence similarity: The DIGS approach relies on sequence similarity searches, which may not always accurately identify novel genome features. There is a possibility of false positive or false negative results.

b. Limited to known sequences: The DIGS approach is limited to identifying EVEs that have some resemblance to previously described sequences. It may miss entirely novel or highly divergent EVEs that do not show significant sequence similarity to known ones.

c. Size limitation.

d. The approach may be limited for identifying recently integrated EVEs. How does the approach work for recently integrated viral sequences in primates?

Overall, this article discusses the power of linking a relational database management system to a similarity search-based screening pipeline for in silico exploration of the dark genome. The DIGS approach used in the study provides a valuable tool for exploring the dark genome and identifying EVEs, but it has its limitations in terms of relying on sequence similarity and potentially missing novel or highly divergent EVEs.

The questions asked in this study include

(i) How many EVE sequences were identified using the database-integrated genome screening (DIGS) approach? Using the DIGS approach, developed by the authors, approximately 1.1 million endogenous viral element (EVE) sequences were identified.

(ii) Were there any previously unreported EVEs discovered in the study? The study identified a set of clearly novel endogenous viral elements (EVEs) that had not been reported before. These novel EVEs were defined as either deriving from a virus group not previously reported in the host group or occurring in species distantly related to those in which similar EVEs had been identified previously. The study included these novel EVEs in their analysis, expanding our knowledge of virus diversity and distribution among vertebrate hosts.

(iii) What are the main types of EVEs found in vertebrate genomes?

Addressing this question, the study raises several interesting points, and some concerns:

The DIGS approach could be useful to identify EVEs that are conserved in all vertebrate classes - would be worth a paragraph in the discussion.

***We agree and have added some sentences in the discussion addressing this point (p25: lines 1-5).***

Page 12, top: "We also identified and excluded hits that appeared likely to derived from exogenous viruses …". I might have missed, but would be important to have more details on how the Figure 4 was generated? How was external viruses and EVEs were distinguished?

***Thank you for drawing our attention to this possible source of confusion regarding figure 4. We did not mean to imply that the exogenous viruses identified in our study are shown in the figure. Rather, the figure shows exogenous virus distribution data based on a review of virus databases and current literature. We now provide extra detail in the results (p11: lines 3-4) and figure legend describing how the figure was generated.***

Figure 4 has some contradiction with the main text: Page 26: "The absence, or near absence, of many virus groups from our catalogue of vertebrate EVEs is noteworthy. For example, vertebrates are infected with many ssRNA+ve viruses, but EVEs derived from these viruses are extremely rare,…" By contrast, Figure 4 suggests that ssRNA+ve viruses are integrated in mammals and generate EVEs. Flaviviruses are highly interesting, because they are ssRNA+ve viruses, similarly to SARS-Cov viruses, and there has been a recent debate on this very topic - see DOI: 10.1126/science.372.6543.674.

***Thank you for highlighting this point. We now express our point more clearly in the manuscript, which is that though flavivirus EVEs do occur, very few of the many different ssRNA+ve virus families that infect vertebrates are represented as EVEs in vertebrate genomes (p24, lines 11-18). For practical reasons, figure 4 only shows the ssRNA+ve families that have generated some vertebrate EVEs. To avoid confusion, we have modified the figure title to reinforce that only viruses represented by EVEs are shown.***

Page 20, line 15: "Additionally, uncharacterised EVE loci may be hard to recognise due to their being highly degraded or fragmented, or because their exogenous virus counterparts are either unknown or extinct [65, 66]." All EVEs are fragmented or there are others that are better preserved?

***We thank the reviewer for spotting this omission, we have now corrected the text to read “Additionally, some uncharacterised EVE loci may be hard to recognise due to their being highly degraded or fragmented, or because their exogenous virus counterparts are either unknown or extinct”.***

Page 10: "Furthermore, the initial integrated form of a retrovirus genome, called a pro-virus, is typically replication competent and increases in germline copy number can thus occur through reinfection of germ line cells [32]. " To be more precise. Endogenous retroviruses (ERVs) have an evolutionary life cycle when they function as transposable elements and can increase the copy number of the germline without leaving the germline and re-infecting.

***We agree that endogenous retroviruses can increase their germline copy number through mechanisms involving intracellular transposition as well as via reinfection of germline cells. We have updated the text and also added a reference describing evidence for both these mechanisms being involved in ERV copy number expansion in primates.***

Minor:

Page 18: "Notably, within the Mammal-1 group, EVEs identified in mammals indigenous to Southern Hemisphere continents (e.g. marsupials, xenarthrans) group …"

Evolutionary biologist would argue that Marsupial are not mammals.

***We have updated the text to read ‘Notably, within the Mammal-1 group, EVEs identified in host species groups that are indigenous to Southern Hemisphere continents (e.g. marsupials, xenarthrans) cluster basally, whereas EVEs and viruses isolated from ‘Old World’-associated placental mammals occupy a more derived position.’***

Page 28: "DIGS has previously been used to broadly survey the distribution of interferon

stimulated genes in mammals [ref],…" - The reference is missing

***Thank you for highlighting this omission - we have updated the missing reference.***

Figure 4 - There are no abbreviations on the figure. The abbreviations can only be found on Figure 6, but it would be important to add them here as well.

***Thank you for bringing this important point to our attention! We have updated the figure to include the abbreviations.***

**Second round of review**

**Reviewer 3**

I am satisfied with the revision.
